# Why the Long “Horns”? Fine‐Scale Morphology Suggests Tactile Demands Contributed to the Exaggeration of Male Longhorned Beetle Antennae (Coleoptera: Cerambycidae)

**DOI:** 10.1002/ece3.71380

**Published:** 2025-05-08

**Authors:** Rowan L. K. French, Magdalena Kowalewska Groszkowska, Locke Rowe, D. Luke Mahler, Lech Karpiński

**Affiliations:** ^1^ Department of Ecology and Evolutionary Biology University of Toronto Toronto Canada; ^2^ Museum and Institute of Zoology, Polish Academy of Sciences Warszawa Poland

**Keywords:** exaggerated trait, functional morphology, mate finding, sensilla, sensory, sexual dimorphism

## Abstract

Insect antennae are covered in hairlike sensilla that detect diverse environmental cues. Selection on these functions has produced a bewildering variety of antennal forms, including many examples of sexual dimorphism (SD). Antenna length SD is particularly common, but poorly understood, in longhorned beetles (Coleoptera: Cerambycidae). Extremely elongate male antennae may extend the reach of individuals searching for mates, enabling rapid recognition via antennal contact. Alternatively, they may increase sensitivity to airborne pheromones by bearing more olfactory sensilla. We tested these hypotheses by modeling sensillum distributions and abundances across species and sexes of *Anoplistes,* a cerambycid genus with extensive variation in antenna length and SD. We found limited evidence that olfactory sensillum abundance scales with antenna segment length; instead, mechano‐ and contact chemosensory sensilla cluster near the antenna tip, consistent with contact‐mediated mate recognition. If the tip segment serves an important tactile role, that may explain why it is exceptionally elongated in males of several species with long, sexually dimorphic antennae. In other *Anoplistes* species with strong antennal SD, however, all segments exhibit similar levels of dimorphism. Collectively, our results suggest that alternative pathways to antenna SD evolved rapidly in *Anoplistes*, perhaps due to different patterns of selection on tactile sensation.

## Graphical abstract

Exceptionally elongated antennae are common in male longhorned beetles (Coleoptera: Cerambycidae), but their function and evolution are poorly understood. Through scanning electron microscopy and antennal morphometrics of multiple *Anoplistes* species (Cerambycidae), our study revealed that elongate antennae are more likely adaptive for close‐range recognition of mates than for long‐range sensitivity to pheromones. We also found two distinct patterns of antennal sexual dimorphism in our focal genus, suggesting that different pathways to antennal dimorphism may evolve over short timescales.

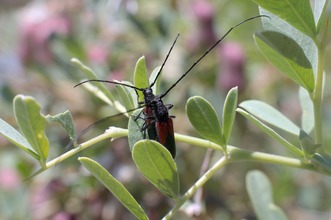

## 1 Introduction

For most insects, antennae are vital to finding food, tracking mates, and recognizing kin. Selection on these and other antenna functions has given rise to an exceptional diversity of antenna forms (Elgar et al. 2018, 2019; Dürr et al. 2022). One prominent axis of antenna diversity is sexual dimorphism in the size, shape, and/or number of segments in the antennae—traits that tend to be larger or more elaborate in males than in females. A common explanation for this pattern is that exaggerated male antennae enhance sensitivity to airborne chemical cues (Schneider 1964; Holwell et al. 2007; Jayaweera and Barry 2017; Johnson et al. 2017; Elgar et al. 2018, 2019; Jaffar‐Bandjee et al. 2020; Pacheco et al. 2024). For species that use sex pheromones to track mates, this “olfactory sensitivity” hypothesis is intuitive; in these species, males with elaborate or elongated antennae detect pheromones produced by females with simpler or shorter antennae (Schneider 1964; Symonds et al. 2011; Ramsey et al. 2015). Here, exaggerated antennae provide a key advantage to males searching for mates: their greater surface area allows them to bear more sensory receptors, or sensilla, which are innervated by sensory neurons that receive stimuli and transmit related cues to the brain (Schneider 1964; Chapman 1982). In taxa reliant on sex pheromones, the branched or elongated antennae of males are typically covered with densely packed olfactory hairs sensitive to long‐range pheromones (Schneider 1964; Elgar et al. 2018, 2019). This dense packing has functional consequences, as higher sensillum abundances are associated with greater responsiveness to airborne cues (Chapman 1982; Spaethe et al. 2007; Jayaweera and Barry 2017; Johnson et al. 2017).

Although many prominent examples of antenna dimorphism seem to have evolved under selection on olfactory sensitivity, there are also numerous counterexamples that do not align with this hypothesis. A key feature of these nonconforming taxa is that they do not display clear sex differences in long‐range attraction to volatile pheromones, despite their sexually dimorphic antennae. In some cases, these taxa do not appear to use airborne pheromones at all (e.g., water striders (Gerridae); Millar 2005); in others, one sex produces a volatile aggregation‐sex pheromone that attracts both sexes (e.g., many cerambycid beetles; Hanks and Millar 2016), suggesting selection on pheromone detection is an unlikely cause of dimorphism. The presence of antenna dimorphism in these taxa suggests that selection on elongation is acting on a function other than olfactory sensitivity. Consistent with this, there are examples of elaborate male antennae that serve nonolfactory functions in courtship (e.g., the coilable antennae of diplazontine wasps; Klopfstein et al. 2010) or coercion during mating (e.g., the hooked antennae of water striders; Khila et al. 2012). However, the shape and/or ultrastructure of those antennae is highly specialized and unique to specific clades. A more common form of antenna dimorphism is a sex difference in the length of a simple, unbranched antenna, with males typically having longer antennae than females (Elgar et al. 2018, 2019). Some non‐sensory functions for elongated male antennae have been proposed, including the use of long antennae as whips or fencing foils during agonistic encounters with other males (Hughes 1981; Hofmann and Schildberger 2001). Another possibility is that selection exaggerates male antennae by acting on a sensory function other than olfaction. Aside from olfactory receptors, antennae host numerous morphologically and functionally distinct sensillum types (Schneider 1964; Staudacher et al. 2005; Dürr et al. 2022). These include hygroreceptors detecting humidity, mechanoreceptors detecting motion or contact, and gustatory receptors detecting non‐volatile chemicals via direct contact. If the antennae are adaptive as mechanical or gustatory “feelers” in one sex, extreme antenna lengths may be favored in that sex (Schneider 1964; Hanks et al. 1996a).

This explanation for antenna length dimorphism, which we refer to as the “antennal reach” hypothesis, has been proposed for the longhorned beetles (Coleoptera: Cerambycidae) (Hanks et al. 1996a). Within this group of more than 35,000 species (Tavakilian and Chevillotte 2023), there are numerous examples of unbranched (filiform) antennae that are extremely long and sexually dimorphic, with male antennae sometimes being more than five times longer than the body and more than two times longer than female antennae (Bezark 2024; Bousquet et al. 2017; Hoskovec et al. 2024; Roguet 2024). It seems unlikely that this variation is due solely to selection on olfactory sensitivity; in contrast to moths, species in the two largest cerambycid subfamilies—Lamiinae Latreille, 1825 and Cerambycinae Latreille, 1802—use male‐produced aggregation‐sex pheromones that attract both sexes (Allison et al. 2004; Hanks and Millar 2016). There is, however, support for the antennal reach hypothesis from empirical work on one cerambycid species with strongly dimorphic antennae, *Phoracantha semipunctata* (Fabricius, 1775) (Hanks et al. 1996a, 1996b). In that species, males actively search for females by walking on host plants with their antennae outstretched. Because mate recognition involves direct contact between the tip of a male's antenna and the body of a female, males with longer antennae may be able to search host plants more effectively and find females more efficiently (Hanks et al. 1996b).

We can test whether the antennal reach hypothesis applies to other cerambycids by examining the abundance and distribution of antennal sensilla across species that vary in antenna length. In species with long, flexible antennae, the most distal segment is often the first to contact a substrate or potential mate (Dürr et al. 2022). Moreover, sensilla are often clustered at the tips of the antennae in species that rely on tactile cues for food‐finding or kin recognition (e.g., katydids—Schneider and Römer 2016; cockroaches—Norris and Chu 1974; ants—Nakanishi et al. 2009). Thus, we might expect sensilla to be more abundant near the antenna's tip than near its base if the antennal reach hypothesis is supported. Conversely, antennal sensilla do not tend to cluster near the antenna tip in species where antennal function suggests support for the olfactory sensitivity hypothesis (Ramsey et al. 2015; Roh et al. 2016; Jayaweera and Barry 2017). Instead, males of taxa that rely on long‐range pheromones tend to have high sensillum abundances across the antenna, due to their enlarged antenna surface areas and/or dense packing of sensilla (Chapman 1982). Sensillum abundance should, therefore, show a strong positive relationship with antenna length under the olfactory sensitivity hypothesis, because the function of elongation is to increase sensillum number. This hypothesis is not necessarily incompatible with the antennal reach hypothesis, as elongated antennae may bear more olfactory sensilla (providing greater olfactory sensitivity) while also allowing males to efficiently sweep their habitats for nearby females. However, the absence of an increase in sensillum number with increasing antenna length would contradict the olfactory sensitivity hypothesis. The antennal reach hypothesis differs in that it does not require sensillum abundance to scale with antenna length; instead, it predicts that mechanosensory and/or contact chemosensory sensilla should be clustered near the antenna tip.

We might gain additional insights into the evolution and development of exaggerated, sexually dimorphic cerambycid antennae by comparing the relative lengths of individual antenna segments. Most cerambycids have unbranched antennae consisting of a muscularized scape, a pedicel involved in proprioception (detection of antenna movement), and a flagellum divided into segments called flagellomeres (A. Minelli 2004, 2017). The flagellum is the primary sensory section of the antenna, and its length varies markedly across Cerambycidae. Some of this variation may stem from among‐species differences in the number of flagellomeres, which ranges from 6 to more than 30 (Santos‐Silva et al. 2016). However, most cerambycids have only nine flagellomeres (the presumed ancestral pattern for beetles; A. Minelli 2004, 2017; Nunes et al. 2020), and most of the antenna length variation seems to occur among such species. Thus, the development of elongate antennae in most cerambycids must require the elongation of individual segments or groups of segments, as opposed to the addition of segments. We do not yet know which segments contribute most to antenna length variation in Cerambycidae, but such data can allow us to ask whether exaggerated antennae have evolved the same way across species—that is, via elongation of the same sets of segments in different taxa. Under the antennal reach hypothesis (but not necessarily the olfactory sensitivity hypothesis), we might expect the most distal antennal segment to be especially elongated in males, as this segment plays an important tactile role in species with long, filiform antennae (Dürr et al. 2022). Increasing the surface area of this segment may allow it to bear more mechanosensory and/or contact chemosensory sensilla, enhancing its tactile sensitivity.

**FIGURE 1:**
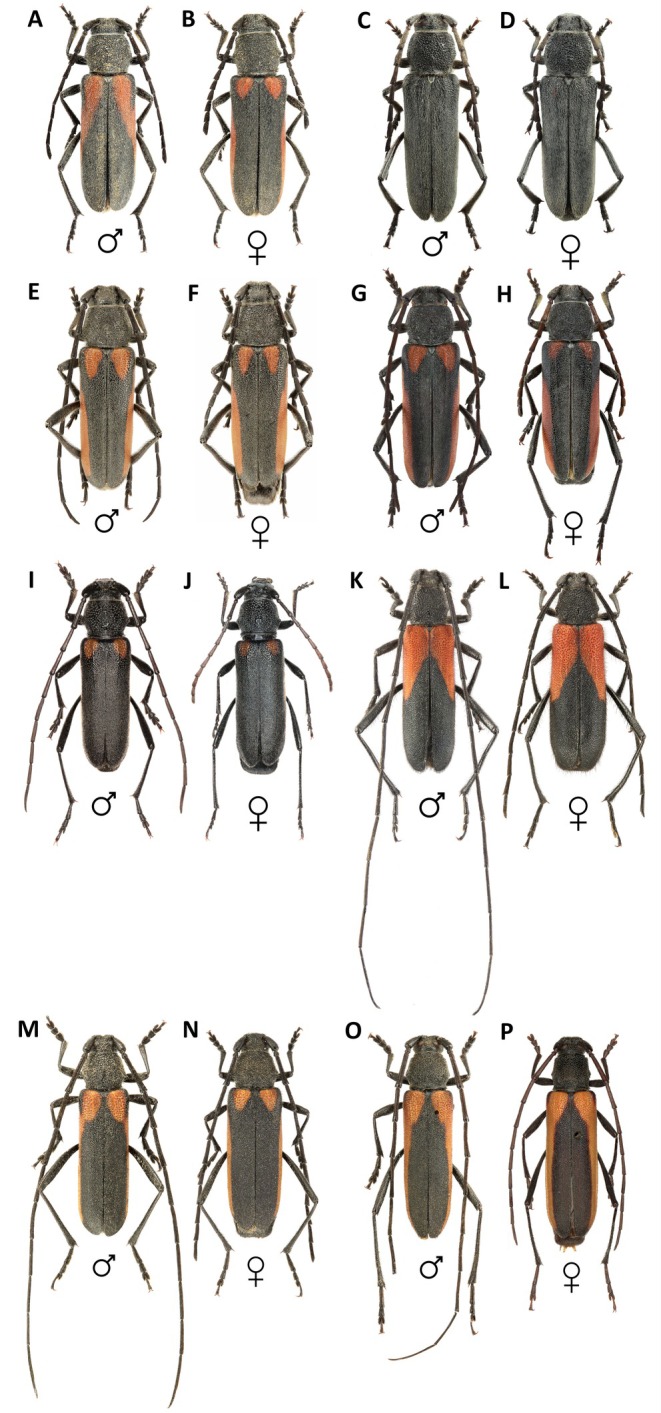
Habitus of the studied *Anoplistes* taxa (A, B): *Anoplistes forticornis* (male and female); (C, D): *Anoplistes galusoi* (male and female); (E, F): *Anoplistes agababiani* (male and female); (G, H): *Anoplistes mongolicus* (male and female); (I, J): *Anoplistes tuvensis* (male and female); (K, L): *Anoplistes jacobsoni* (male and female); (M, N): *Anoplistes halodendri halodendri* (male and female; rock ecotype); (O, P): *Anoplistes halodendri ephippium* (male and female).

**FIGURE 2:**
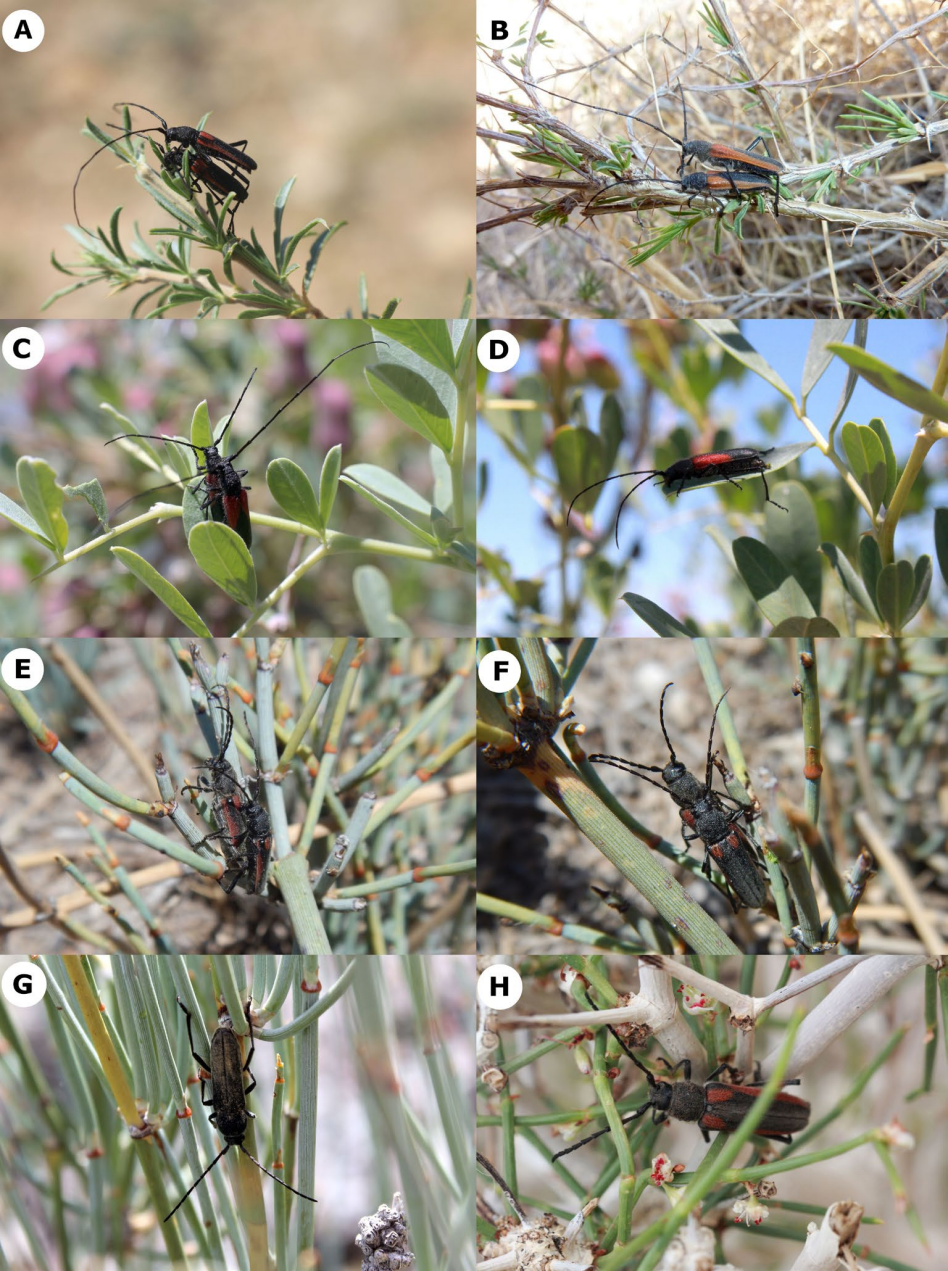
Photographs of adult beetles in situ (A) *Anoplistes halodendri halodendri* (rock ecotype) in copula on *Caragana*; (B) *Anoplistes halodendri halodendri* (sand ecotype) in copula on *Caragana*; (C, D) *Anoplistes jacobsoni* in copula on *Halimodendron* (C), solitary female (D); (E, F) *Anoplistes forticornis* in copula on *Ephedra*; (G) *Anoplistes galusoi* male on *Ephedra*; (H) *Anoplistes mongolicus* female.

**FIGURE 3:**
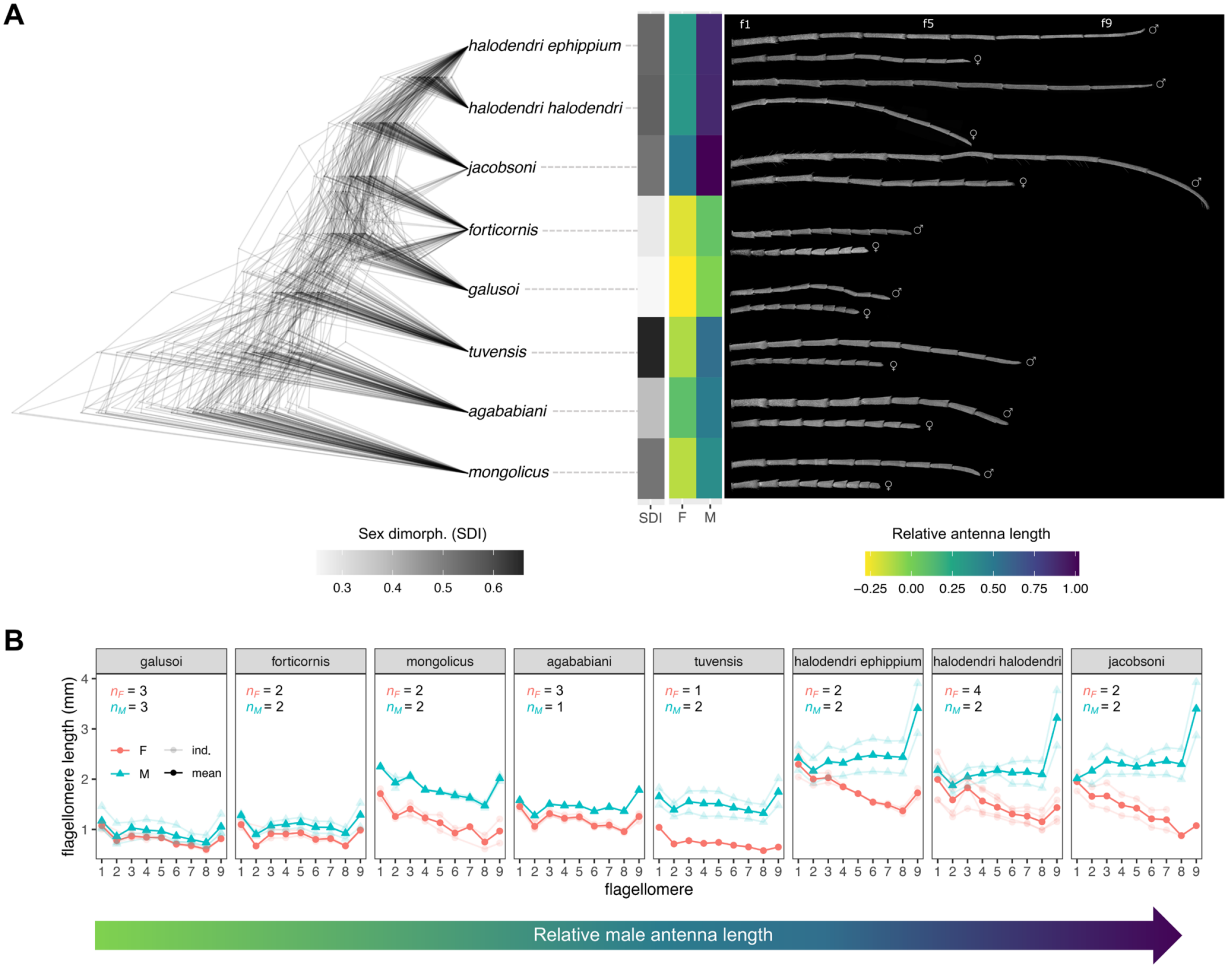
Antennal forms in *Anoplistes* (A) Phylogenetic variation in antenna length and sexual dimorphism. Left: The 100 phylogenies we used for statistical analyses, plotted using densiTree. Middle: Columns representing mean antenna traits for each (sub‐) species. Colored grid cells show relative antenna lengths—that is, log ratios of antennal length and elytral length (a body size proxy) for females (middle column) or males (right column). Higher values (darker purples) indicate longer antennae, relative to body size. Sexual dimorphism in relative antenna length (male relative antenna length—female relative antenna length) is plotted in greyscale (left column); darker grid cells indicate that males have longer antennae than females, after accounting for body size. Right: Composite SEMs of male and female antennae for each of the 8 focal taxa, with antennae scaled proportional to the ratio of antenna length divided by elytron length (B) Flagellomere length (mm) plotted against flagellomere identity. Flagellomere 9 is the most distal antenna segment. Females are pink; males are blue. Sex and taxon‐specific means are plotted (triangles, circles, and lines with no transparency) along with raw, individual‐level data (partial transparency). Due to missing antennal segments or deformities, some individuals do not have length data for all segments. Plots are arranged according to relative male antenna length, from the taxon with the lowest value (*A. galusoi*) to the one with the highest ( *A. jacobsoni* ). Sample sizes for males and females are provided for each plot. The individuals used in (B) were also used in downstream brms models of sensillum abundance and density.

The Palaearctic cerambycid genus *Anoplistes* Audinet‐Serville, 1833 (Cerambycidae: Cerambycinae: Trachyderini) is ideally suited to testing the olfactory sensitivity and antennal reach hypotheses and examining variation in antennal flagellomere lengths. From a prior description of antennal sensilla in *Anoplistes halodendri* (Pallas, 1773), we know that at least one member of this genus has putative contact chemoreceptors and olfactory receptors (Liu et al. 2012). Thus, *A. halodendri* seems to resemble other cerambycids that respond to both airborne signals (host plant volatiles and/or airborne pheromones) and contact chemical cues (e.g., *Monochamus* species—Dyer and Seabrook 1975; Ibeas et al. 2009; Pajares et al. 2010; Huh et al. 2023). Aside from *A. halodendri*, the genus contains 13 other described species and several subspecies of its type species (Karpiński 2020; Karpiński et al. 2021; Tavakilian and Chevillotte 2023). Most of these taxa are distributed in poorly explored arid regions in Central Asia (Figure S1), and available ecological, morphological, and genetic data suggest that several species within this group diverged relatively recently—approximately 10 Mya or less (Karpiński et al. 2021, 2023). There is substantial variation in both antenna length and sexual dimorphism across members of the genus (Figures 1, 2, 3), making it an ideal taxon for this study. So prominent is this variation across *Anoplistes* that taxonomists have historically sorted some members of this genus into “short” and “long” antennal groups based on antenna lengths and levels of sexual dimorphism (Plavilstshikov 1940; Kostin 1974). However, both traits vary continuously among species, and there are several intermediate species that cannot be easily sorted into either antennal group. All *Anoplistes* antennae have nine flagellomeres, like most members of the Cerambycidae.

Here, we leverage the exceptional antenna length diversity of *Anoplistes* to test two functional hypotheses about elongate antennae: olfactory sensitivity and antennal reach. To do this, we imaged antennal sensilla in eight *Anoplistes* taxa, then examined how sex, flagellomere length, and flagellomere identity influence sensillum abundance and density across taxa. In addition, we asked which segments contribute most to antennal exaggeration/sexual dimorphism by comparing the lengths of individual antenna segments among sexes and taxa. These comparisons allowed us to test our “antennal reach” prediction that the most distal segment should be exaggerated in males of species with strongly dimorphic antennae.

## 2 Materials and Methods

### 2.1 Material Examined

Within *Anoplistes*, we focused on eight taxa (seven species) that, together, represent the range of variation in antenna length and dimorphism seen across the genus (Figure 1). We sampled three taxa from the “long‐antennae” *Anoplistes halodendri* species‐group (Plavilstshikov 1940; Kostin 1974; Karpiński 2020): *Anoplistes halodendri halodendri* (Pallas, 1773), *Anoplistes halodendri ephippium* (Steven and Dalman, 1817), and *A. jacobsoni* Baeckmann, 1904. We also sampled two species ( *A. forticornis* Reitter, 1901 and *A*. *galusoi* (Kostin 1974)) from the “short‐antennae” *Anoplistes forticornis* species‐group (Plavilstshikov 1940; Kostin 1974; Karpiński et al. 2023). Finally, we included three intermediate species: *A. mongolicus* Ganglbauer, 1889, *A*. *agababiani* (Danilevsky, 2000), and *A. tuvensis* (Tsherepanov, 1978). Regarding *A*. *halodendri*, we chose to separately analyze two subspecies of this species because several lines of evidence indicate that *A. h. ephippium* and *A. h. halodendri* are independently evolving lineages (see Discussion).

Most of the specimens used in this study were collected by one of this study's authors, L.K. (MIZ—Museum and Institute of Zoology, Polish Academy of Sciences, Warszawa, Poland) or his collaborators during expeditions to Kazakhstan, Kyrgyzstan, and Mongolia. The remaining specimens were loaned from museum and private collections: CCH—Collection of Carolus Holzschuh (Villach, Austria); HNHM—Hungarian Natural History Museum (Budapest, Hungary); MAS—Institute of Biology, Mongolian Academy of Sciences (Ulaanbaatar, Mongolia); MNHN—Museum national d'Histoire naturelle (Paris, France); NHMUK—Natural History Museum (London, UK); NMP—National Museum Prague (Prague, Czech Republic); ZIN—Zoological Institute, Russian Academy of Sciences (St. Petersburg, Russia).

### 2.2 Phylogenetic Data

To account for phylogenetic effects in our downstream analyses, we needed an ultrametric phylogeny including all eight focal taxa. We opted for a combined morphological and molecular approach to phylogenetic reconstruction, as molecular sequences or molecular‐grade specimens were not available for two of our focal species ( *A. mongolicus* and *A. agababiani*). As the outgroup for our tree, we chose the closely related species *Purpuricenus kaehleri* (Linnaeus, 1758), following Karpiński et al. (2021). We scored 13 categorical morphological characters for all taxa, using specimens loaned from museums and private collections (Tables S1 and S2). All ingroup (*Anoplistes*) taxa had complete coverage for these characters; however, we were unable to score one character in our outgroup. In addition to scoring morphological characters, we leveraged sequences of the 658 base‐pair “barcode” region of cytochrome c oxidase subunit I (COI) from GenBank that had been generated by L.K. for previous publications (Karpiński et al. 2021, 2023). In total, we used 29 previously published sequences for five of our ingroup taxa (1–17 sequences per taxon) and one sequence for the outgroup species. Analyzing multiple sequences per taxon allowed us to test the monophyly of individual species. Although other gene sequences were available for a small number of our focal taxa (1 or 2 species per gene), we chose to focus on COI because it had the greatest number of publicly available sequences, showed the best coverage across our focal taxa, and has previously been used to reconstruct phylogenetic relationships among *Anoplistes* species (Karpiński et al. 2021, 2023). We increased our species‐level sampling of COI by sequencing the “barcode” region of this gene from one specimen of a sixth taxon: *A. tuvensis* (GenBank accession #: PQ677796). This specimen was killed using ethyl acetate and later placed in 99% ethanol and stored in an ultralow freezer. Prior to sequencing, we extracted DNA from leg tissue using a phenol‐chloroform method, amplified COI following established protocols (Hebert et al. 2003), cleaned up the reaction using an ExoSAP‐IT kit, and performed Sanger sequencing at the University of Alberta's Molecular Biology Services Unit. We then filtered, aligned, and trimmed our COI sequence dataset using the command‐line SuperCRUNCH pipeline (Portik and Wiens 2020). To quality‐filter sequences, we first downloaded nine Cerambycidae species' mitochondrial genomes from GenBank and extracted high‐quality “reference” COI sequences from these genomes (see supplementary data on Zenodo). Next, we used the SuperCRUNCH script called “Reference_Blast_Extract.py” to create a custom BLAST database for comparing reference and target COI sequences. After this filtering step, target sequences were trimmed using the gt algorithm in trimAl, as implemented in the “Trim_Alignments_Trimal.py” SuperCRUNCH script (Capella‐Gutiérrez et al. 2009). We used default settings for all other parts of the SuperCRUNCH pipeline. After running SuperCRUNCH, we concatenated the trimmed, 658 bp molecular matrix and our morphological matrix using Mesquite 3.81 (Maddison and Maddison 2023).

### 2.3 Scanning Electron Microscopy

We used a scanning electron microscope (SEM) to image individual flagellomeres and antennal sensilla. Here, our sampling consisted of 16 male and 19 female specimens (1–4 specimens per sex and taxon) loaned from museums and private collections. Prior to SEM, we mounted each whole specimen on an aluminum stub with carbon tape and positioned a randomly selected antenna so that its ventral surface faced upward. We chose to image the ventral side of the antenna because we reasoned that it should be important for both olfaction and tactile sensation, as it can make direct contact with the substrate. Additionally, a previous SEM study of *Anoplistes halodendri* found high sensillum abundances on both the ventral and dorsal sides of the antenna, but the dorsal side was dominated by putative mechanosensory sensilla (sensilla chaetica), while the ventral side bore more diverse sensillum types (Liu et al. 2012). Observations of antennal sensilla were made using an SEM (Hitachi S‐3400N) in the SEM Laboratory of the Museum and Institute of Zoology of the Polish Academy of Sciences (Warsaw). We used this microscope's low vacuum mode because it did not require specimens to be fixed, dehydrated, or sputter‐coated prior to analysis. The SEM was operated at 20 keV and a working distance of 10 mm. For each sex and taxon, we scanned all nine flagellomeres, omitting the scape and pedicel (the two most proximal antenna segments) because these do not have a major sensory function in insects. We took multiple images of each flagellomere, including one whole‐segment image (magnification: 35×–100×) and 3–5 higher magnification images (150×–1700×) of random subregions of the flagellomere. Whole‐segment images were used to measure the lengths and widths of individual segments, and to estimate total sensillum abundances and the abundances of long chemosensory sensilla (as described in Section 2.5); higher magnification images were used to quantify the abundances of other subtypes of sensilla (see Section 2.5 for details).

### 2.4 Morphometric Data

Using imageJ 1.53 (Schindelin et al. 2015), we measured antennal flagellum lengths (hereafter “antenna lengths”) and elytron lengths (our body size proxy) from dorsal images of our eight focal *Anoplistes* taxa (27 males, 23 females; 1–8 specimens per sex and taxon). These images include photographs of museum specimens taken by us (see supplement for details), images from published papers (Karpiński 2020; Karpiński et al. 2021, 2023), and photographs downloaded from species pages on the “Longhorn Beetles of the West Palearctic Region” website (Hoskovec et al. 2024). We reduced the risk of measuring the same specimen multiple times by carefully inspecting images from different sources (i.e., to ensure that photographs were not re‐used among sources), and by comparing specimen label data (when available). As a measure of relative antenna length, we calculated the log ratio of each specimen's antenna length to its elytron length. We averaged these values for each taxon and sex, then estimated an index of sexual dimorphism for each taxon by subtracting the mean relative antenna length for females from the mean relative antenna length for males, following Adams et al. (2020). Larger positive values of this index indicate an increase in the length of male antennae, relative to female antennae.

To understand how variation in the lengths of individual antenna segments contribute to among‐species and between‐sex differences in total antenna length, we measured the lengths of flagellomeres 1–9 from SEM images of whole segments (306 segments in total; *n* = 1–4 per flagellomere type, sex, and taxon). For comparison with segment length patterns, we also measured segment width in the middle of each segment. Each image was scaled prior to measurement using its inset scale bar, and all measurements were made in imageJ.

### 2.5 Sensillum Data

To distinguish between our two hypotheses about the function of exaggerated antennae, we needed estimates of sensillum abundance across the ventral surface of different flagellomeres. We used imageJ to collect these data, focusing on the first, fifth, and ninth flagellomeres (f1, f5, and f9), as these segments represent three (potentially) functionally distinct regions of the antennal flagellum: the base, middle, and tip. To estimate the number of sensilla on each of those segments, we overlaid a square grid on each image, defining the width and length of each grid box (quadrat) as half the width of the flagellomere. We then chose three boxes that fully overlapped the flagellomere—one near the proximal end, one in the middle, and one near the distal end. We counted the total number of sensilla in each of these boxes, calculated within‐box density based on the area of each box, then averaged the three within‐box densities to estimate the mean sensillum density across the whole ventral surface of the flagellomere. To understand whether our mean estimates were skewed by outliers (given our low sampling of quadrats), we plotted the relationship between mean and median sensillum density across individuals and calculated the Pearson correlation coefficient for each flagellomere. Means and medians were highly correlated in all cases (f1 = 0.982, f5 = 0.986, f9 = 0.963; Figure S2), so we present results based on mean sensillum densities. Finally, to estimate sensillum abundance for the aspect of the flagellomere visible in each photograph, we multiplied our mean density estimate by the visible two‐dimensional area of the flagellomere. Areas were measured by tracing the flagellomere's outline with the polygon tool in ImageJ. In total, we analyzed 101 flagellomeres (*n* = 1–4 per flagellomere type, sex, and taxon).

In addition to collecting data on total sensillum abundance, we were interested in understanding (1) whether species, sexes, or flagellomeres differ qualitatively in the functional types or distributions of sensilla they possess and (2) how the abundances of mechanosensory and chemosensory sensilla vary in relation to flagellomere length. To answer the first question, we used our SEM images and a recent review of cerambycid sensillum types (Haddad et al. 2023) to identify putative mechanosensory and chemosensory (i.e., olfactory and gustatory) sensilla on each of the nine flagellomeres. In brief, we identified sensilla based on their size, shape, sheath texture (grooved or smooth), and socket morphology. We could not score whether sensilla had pores (a feature that is diagnostic of chemosensory sensilla) due to resolution limitations. However, we were able to score other traits (described below) that, in combination, are characteristic of sensilla with probable chemosensory and mechanosensory functions.

Along the edges of f9 (the tip segment), we found one sensillum type that was blunt‐tipped and upward‐curving, resembling either chemosensory wall pore hairs, which are olfactory, or terminal pore hairs, which are gustatory (Haddad et al. 2023). Collectively, we refer to these as “long chemosensory hairs”. These hairs appeared to be clustered near the tip of the last antenna segment, as might be expected if the antenna tip serves an important contact chemosensory function. To test this, we divided the last antenna segment into an “apical half” closer to the tip and a “basal half” and counted the number of long chemosensory hairs visible along the upper edge of the segment within each half.

Two other potentially functionally important morphotypes of sensilla were abundant and identifiable on the surfaces of many segments. These were (1) mechanosensory chaetic sensilla, which have grooved walls and sharply pointed tips (Haddad et al. 2023), and (2) short, conical sensilla that we refer to as “short olfactory hairs”. The latter class includes putative sensilla basiconica and sensilla auricillica (Haddad et al. 2023). We grouped them together because they are functionally similar (in that they both detect long‐range volatile chemicals; Haddad et al. 2023) and were morphologically challenging to distinguish in some of our images. To estimate the abundances of mechanosensory and short olfactory sensilla, we used our higher magnification images of f9. For each image, we overlaid a 40 μm × 40 μm grid, chose a box in the upper left corner, and counted mechanosensory and short olfactory hairs. When images were taken at lower magnifications (i.e., closer to 150× than 1700×) and showed more than ~20% of a segment's surface, we also counted sensilla in a second quadrat in the bottom‐right corner of the image. Like we did for total sensillum abundance, we used our count data (averaged across boxes) to estimate the abundance of each sensillum type on the ventral surface of each specimen's f9, then we calculated a mean for each taxon and sex.

### 2.6 Phylogenetic Analyses

To estimate phylogenetic relationships among our focal *Anoplistes* taxa, we first conducted a Bayesian phylogenetic analysis in MrBayes 3.2.7a (Huelsenbeck and Ronquist 2001), using separate partitions for the morphological and molecular data. For the morphological dataset, we assumed a gamma rate distribution; for COI, we used a generalized time reversible (GTR) model and a gamma rate distribution with a proportion of invariable sites. Evolutionary rates were allowed to vary among partitions. The analysis ran for 50 million MCMC generations in four independent runs with four chains each, sampling one tree per 5000 trees generated. We then summarized our results as a 50% majority‐rule consensus tree (hereafter our “total evidence tree”), after removing the first 25% of MCMC samples from each run as burn‐in. All other parameters were left at default settings.

Our total evidence analysis allowed us to estimate the phylogenetic placement of two species without COI data: *Anoplistes agababiani* and *A. mongolicus* . However, branch lengths were challenging to interpret in this tree, as our analyses were based on mixed morphological and molecular data and we lacked external calibration information (e.g., fossils). To infer both a topology and interpretable branch lengths in units of relative time for the species with molecular data, we separately analyzed our molecular (COI) alignment under an optimized relaxed clock model in the Bayesian program BEAST 2.7.6 (Drummond et al. 2006; Bouckaert et al. 2019; Zhang and Drummond 2020; Douglas et al. 2021). We ran this analysis on a reduced alignment consisting of only 7 sequences, each representing the longest available COI sequence for the (sub‐)species in our ingroup (6 sequences, representing all taxa except *A. agababiani* and *A. mongolicus* ) and outgroup (1 sequence). When running BEAST, we used a GTR model and estimated the proportion of invariant sites. The analyses ran for 150 million MCMC generations, sampling every 15,000 trees. We tested for convergence by running the analysis four times using different randomly selected starting trees.

After running BEAST four times, we removed the first 25% of trees from each run file using LogCombiner (Drummond and Rambaut 2007) and summarized the remaining output by generating a maximum clade credibility (mcc) tree in TreeAnnotator (Drummond and Rambaut 2007). Branch lengths represent common ancestor heights and were averaged across all trees in which a particular clade appeared. All MrBayes and BEAST analyses were performed in CIPRES (Miller et al. 2010), and we assessed convergence and sampling quality by examining effective sample sizes and trace plots in Tracer (Rambaut et al. 2018). We plotted both the total evidence consensus tree and the mcc tree using FigTree v1.4.4 (Rambaut 2018).

Our consensus and mcc trees are useful as visual summaries of phylogenetic relationships, but each tree had some poorly supported nodes. Thus, it was important to account for phylogenetic uncertainty in our downstream statistical analyses of sensillum traits. We did this by sampling 100 trees from the posterior of our BEAST analysis of the COI‐only alignment. To add *A. mongolicus* and *A. agababiani* to each tree, we used the bind.tip function from the phytools package (Revell 2024) in R. Informed by the topology of our total evidence tree from MrBayes, we added both taxa to randomly selected positions along the internode between our outgroup and the remaining *Anoplistes* taxa, with *A. mongolicus* grafted closer to the root than *A. agababiani*. We ran all downstream statistical models iteratively on each of our 100 trees, allowing us to account for variation in both tree topology and branch lengths. To visualize differences in the topologies of these 100 trees, we overlaid them using the densiTree function in the phangorn R package (Bouckaert 2010; Schliep 2011).

### 2.7 Morphometric Analyses

To visualize the phylogenetic distribution of our three focal antenna traits, we plotted species means for relative male and female antenna lengths and sexual dimorphism indices (SDI) using ggplot2 (Wickham 2016). Then, to estimate phylogenetic signal for relative antenna lengths and sexual dimorphism, we used the phylosig function from phytools to calculate Pagel's lambda for each tree in our sample of 100 phylogenies. We summarized the results by estimating a mean and standard deviation for lambda across the 100 trees. A trait with a mean lambda value near 1 shows strong phylogenetic signal, indicating a phylogenetic pattern of trait divergence consistent with a Brownian motion model of evolution.

Additionally, we wanted to determine whether particular flagellomeres contributed disproportionately to antenna length variation within and among species. To visually assess this, we created line plots of mean segment length for each sex and species, again using ggplot2. We created similar plots for segment width to provide a comparison for our segment length plots.

Finally, we used our morphometric data to test a key assumption of our olfactory sensitivity hypothesis: that flagellomere length and surface area are positively correlated. We tested this by fitting a Bayesian phylogenetic linear mixed model (LMM) to individual‐level data using the R package brms (Bürkner 2017, 2018). Our response variable was log‐standardized antenna surface area, and our predictors included a three‐way interaction between sex, flagellomere identity (f1, f5, or f9), and scaled, log‐standardized flagellomere length. We also used three types of random intercept effects: a phylogenetic correlation matrix and a nested effect of individual (*n* = 35) within taxa (species or subspecies; *n* = 8). The purpose of these random effects was to account for structure in the residuals due to shared evolutionary history, non‐phylogenetic variation among taxa, and among‐individual differences. Additionally, to account for phylogenetic uncertainty, we ran this model (and all sensillum models described in the next section) 100 times, with each iteration fitted to a different phylogenetic correlation matrix (each corresponding to one of the 100 samples from the posterior distribution of our BEAST analysis with *A. mongolicus* and *A. agababiani* grafted in). We summarized our results by pooling posteriors across iterations using the combine_models function in brms. For this model and all brms models described below, we used trace plots to confirm that our model chains mixed appropriately and converged. We also confirmed that data simulated under our model did not systematically differ from our observed response data by performing posterior predictive checks (Figure S3). Our final segment area model (and all models described in the next section) passed this preliminary visual diagnostic test, as the observed response data consistently fell within the range of our simulated data.

To visualize our model results, we used ggplot2. Specifically, we plotted observed means for log flagellomere length (*x*‐axis) and log segment area (*y*‐axis) for each species and sex, with plots faceted by flagellomere. Atop our observed data, we plotted curves and ribbons representing the model‐estimated conditional effect and 95% credibility interval (CI) of log flagellomere length on log flagellomere area. Each curve (and its corresponding ribbon) represents results for a different sex and flagellomere, based on model results pooled across 100 trees. We extracted each conditional effect and the bounds of its 95% CI using the make_conditions and conditional_effects functions from the brms package. Additionally, we generated separate plots of the slope parameter estimates from our models using the mcmc_intervals function from the brms package. Here and in the brms models described below, we interpreted a parameter as affecting our response variable if its 95% CI did not include zero. All analyses and plots described in this section and the next section were performed or created in R 4.3.1 (R Core Team 2023).

### 2.8 Sensillum Analyses

To test whether sensilla were clustered near the tips of the antennae or were more abundant on longer antenna segments, we fit Bayesian phylogenetic generalized linear mixed models (GLMMs) to individual‐level data in brms. Our model of total sensillum abundance had the same predictors and random effect structure as our segment area model (see previous section). For this model and all sensillum models described below (unless otherwise noted), we used a negative binomial distribution for each response variable, as our data tended to be overdispersed. For each model, we used default priors for all but the slope parameters, for which we used Gaussian priors with a mean of 0 and a standard deviation of 1, to improve sampling efficiency relative to default flat priors. Additionally, we used 10,000 iterations, an adapt delta value of 0.9999, a step size of 0.01, and a maximum tree depth of 12 for this model and all other models.

To better understand variation in total sensillum abundance, we also modeled sensillum density on f1, f5, and f9 using data from the whole‐segment images. One method of modeling rates like sensillum density is to fit a negative binomial model to count data while including an offset term that accounts for variable areas among sampling units. Here, our response variable was the cumulative abundance of sensilla in the three boxes on each flagellomere (f1, f5, or f9), and our offset term was the log‐standardized total area of those three boxes. The structure of our sensillum density model was otherwise identical to that of our total sensillum abundance model.

To test whether flagellomere length and/or sex predict variation in the abundance of mechanosensory hairs, short olfactory hairs, or long chemosensory hairs on the most distal antenna segment, we ran three separate brms models. Our mechanosensory and short olfactory models had the same structure, with sex, segment length, and their interaction as predictors and taxon and phylogenetic structure as random intercept effects. Our long chemosensory model had a different structure. It included a three‐way interaction between sex, segment length, and within‐segment position (apical half or basal half), allowing us to test whether long chemosensory sensilla were clustered near the tip of the antenna. The random effect structure of the long chemosensory model mirrored that used in our model of total sensillum abundance. Trace plots and posterior predictive plots for the sensillum models are available in the supplement (Figures S4–S8). We separately estimated and plotted model parameter estimates and the conditional effect of log flagellomere length on each sensillum trait using functions from the brms and ggplot2 packages, as described in the previous section.

## 3 Results

### 3.1 Phylogeny

The total evidence tree (MrBayes; morphological and COI data) and the maximum clade credibility tree (BEAST; COI only) generally had congruent topologies, although some nodes had low support in each tree (Figure S9). One key area of uncertainty was in the phylogenetic placement of *A. galusoi*. According to our MrBayes analysis, there was weak support (posterior probability (PP) = 62%) for a clade in which *A. galusoi* and *A. forticornis* were sisters (Figure S9A). The position of *A. galusoi* was also ambiguous across our BEAST trees, with a slight majority (55%) of those trees recovering *A. galusoi* as sister to a clade including *A. halodendri*, *A. jacobsoni* , and *A. forticornis* (Figure S9B). Otherwise, the COI‐only analyses tended to recover more strongly supported species‐level relationships (PP = 0.84–1) than did the total evidence analyses (PP = 0.71–1).

Relative to the COI‐only alignment, the total evidence alignment included two unique species (*A. agababiani* and *A. mongolicus* ) and additional specimens of several other *Anoplistes* (sub‐) species. Our total evidence analyses showed strong support for a topology in which *A. agababiani* and *A. mongolicus* were sister to a clade containing all other *Anoplistes* species (PP = 0.92–1). We also found strong support for the monophyly of all species for which we analyzed multiple specimens (PP = 0.97–1), including *A. forticornis, A. galusoi, A. jacobsoni*, and *A. halodendri*. However, *A. halodendri halodendri* and *A. halodendri ephippium* were not reciprocally monophyletic in our total evidence phylogeny (Figure S9A). Nevertheless, in downstream analyses, we treated *A. halodendri ephippium* and *A. halodendri halodendri* as separate taxa, as there is strong evidence from previously published work and our own field observations that these named subspecies represent geographically isolated, independently evolving lineages (see Discussion).

### 3.2 Variation in Antenna Form

In all eight taxa we examined, males had longer antennae than females after accounting for body size (Figure 3A). However, the degree of sexual dimorphism varied considerably among species, as did relative male and female antenna lengths. We observed the strongest sexual dimorphism in relative antenna length in *A. tuvensis* and the weakest in *A. galusoi* and *A. forticornis* (Figure 3A). The latter two species also had the shortest male and female antennae of the species we sampled, while *A. jacobsoni* had the longest (Figures 1 and 3A). Phylogenetic signal was strong for sexual dimorphism in antenna length (mean lambda = 1.18, std. dev. = 0.19 across 100 phylogenetic trees), relative female antenna lengths (mean lambda = 1.16, std. dev. = 0.23), and relative male antenna lengths (mean lambda = 1.16, std. dev. = 0.23).

In addition to measuring total antenna lengths, we compared the lengths of individual flagellomeres within and between sexes (Figure 3B). These comparisons allowed us to test another prediction of the antennal reach hypothesis: that the most distal antennal segment (the one that should make first contact with a substrate) is the most exaggerated. Our results are consistent with that prediction; across taxa and sexes, the tip segment (f9) tended to be longer than more proximal segments—particularly f8. This pattern was most obvious in males, especially in the three taxa with the longest male antennae: *A. jacobsoni* , *A. halodendri halodendri*, and *A. halodendri ephippium*.

While comparing flagellomere lengths, we also found an unexpected result: there are two distinct patterns of antennal sexual dimorphism in our sample of *Anoplistes* species. In the three taxa with the longest male antennae, sexual dimorphism in segment length increased distally, with f9 being the most dimorphic (Figure 3B). Thus, male and female antennal segments were shaped differently (i.e., showed different length ratios) in those taxa. In all other species, sexual dimorphism in flagellomere length was relatively constant from the base to the tip of the flagellum, with male antennae resembling scaled‐up versions of female antennae.

In contrast to segment length, we did not see clear or consistent patterns of segment width variation among species (Figure S10). However, longer, more distal segments were sometimes narrower—particularly in *A. halodendri* and *A. halodendri ephippium*. Despite this, segment lengths and surface areas were positively correlated across species, sexes, and segments (Gaussian LMM: est.effect = 0.58 ± 0.05; 95% credibility interval: 0.48–0.69) (Figure S11).

### 3.3 Qualitative Variation in Sensilla Along the Length of the Antenna

**FIGURE 4:**
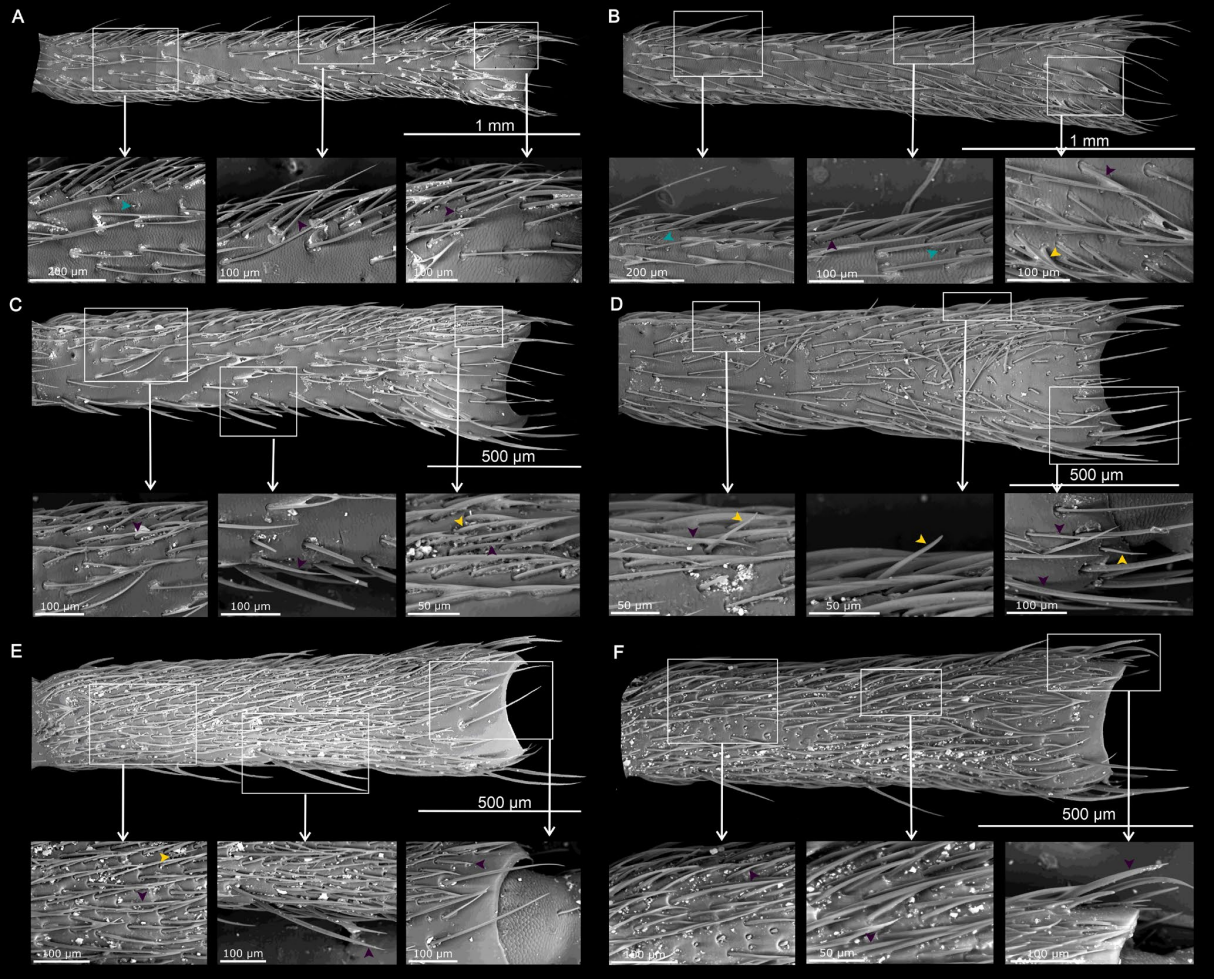
Distribution of sensilla on the most basal segment of the antennal flagellum (f1) of (A, B) *Anoplistes halodendri ephippium*, (C, D) *A. agababiani*, and (E, F) *A. galusoi*. Males are in the left column (A, C, E); females are on the right (B, D, F). Arrows on inset images indicate examples of different sensillum types (teal = short olfactory hairs; yellow = long chemosensory hairs; purple = mechanosensory hairs).

**FIGURE 5:**
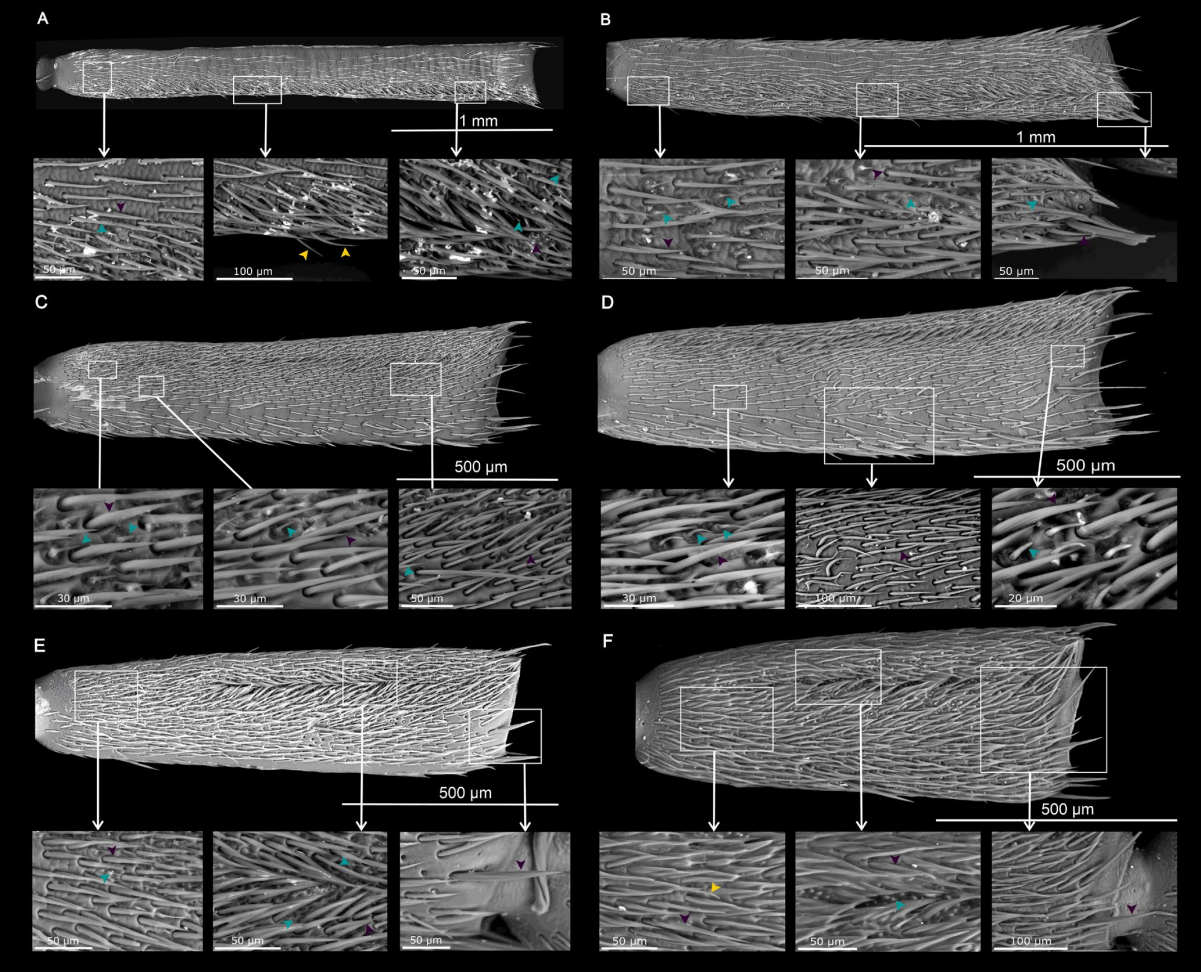
Distribution of sensilla on the segment in the middle of the antennal flagellum (f5) of (A, B) *Anoplistes halodendri ephippium*, (C, D) *A. agababiani*, and (E, F) *A. galusoi*. Males are in the left column (A, C, E); females are on the right (B, D, F). Arrows on inset images indicate examples of different sensillum types (teal = short olfactory hairs; yellow = long chemosensory hairs; purple = mechanosensory hairs).

**FIGURE 6:**
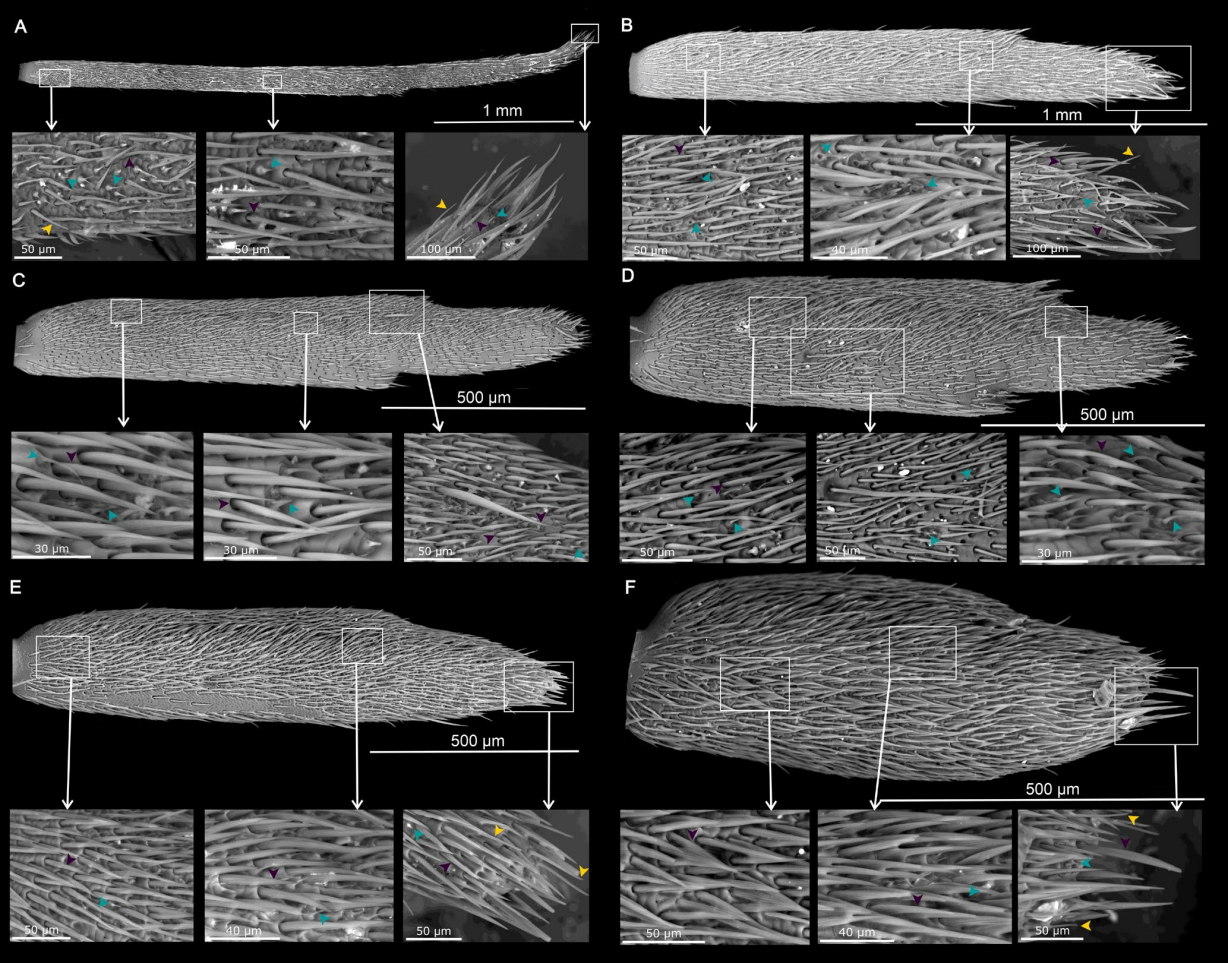
Distribution of sensilla on the tip of the antennal flagellum (f9) of (A, B) *Anoplistes halodendri ephippium*, (C, D) *A. agababiani*, and (E, F) *A. galusoi*. Males are in the left column (A, C, E); females are on the right (B, D, F). Arrows on inset images indicate examples of different sensillum types (teal = short olfactory hairs; yellow = long chemosensory hairs; purple = mechanosensory hairs).

At the scale of our analysis, there were qualitatively similar types and distributions of sensilla across sexes and species (Figures 4, 5, 6). Mechanosensory hairs were the most abundant type of sensillum on the antennae of all *Anoplistes* species, being distributed across the surfaces of all flagellomeres. Short olfactory hairs were the next most abundant sensillum group. Within flagellomeres, these sensilla tended to show a more clumped distribution than mechanosensory hairs, and their abundance was visibly higher near the tip of the antennal flagellum than near the base. The first (most proximal) flagellomere bore no or few short olfactory hairs. On flagellomeres 2–9, these hairs were either confined to or densest in a groove that ran along the length of the segment. They spilled out of these grooves on the more distal segments to form larger sensory fields—particularly on f9. In contrast, long chemosensory hairs were visible on all segments but less abundant than mechanosensory hairs. On the more distal segments (between f5 and f9), long chemosensory hairs were also less abundant than short olfactory hairs (Figures 5 and 6).

### 3.4 Abundance and Distribution of Sensilla

**FIGURE 7:**
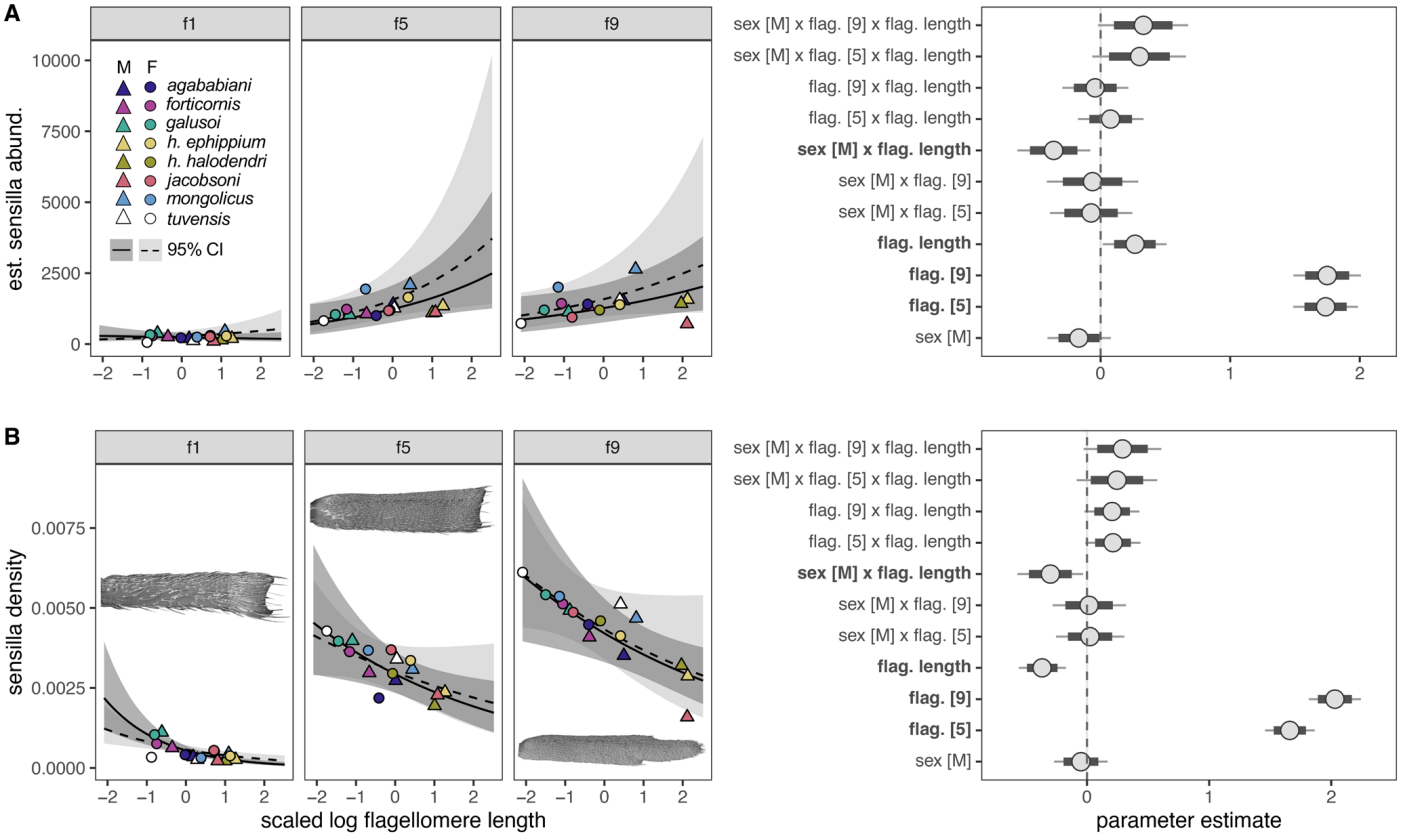
(A) Total sensillum abundance and (B) sensillum density in relation to flagellomere length, sex, and flagellomere identity. Curves and ribbons in the leftmost panels of (A) and (B) represent estimated relationships and 95% credibility intervals for males (dark gray; solid line; triangles) and females (light gray; dashed line; circles) for f1, f5, and f9. Points (triangles and circles) represent means for individual sexes and taxa. Insets in (B) are flagellomeres (f1, f5, and f9) of male *A. agababiani* and show differences in flagellomere shape and sensillum abundance. Parameter estimates from brms are shown in the rightmost panel of (A) and (B). Circles represent means, thick lines represent 80% credibility intervals (CIs), and thin lines represent 95% CIs. Parameters with 95% CIs that do not overlap 0 affect sensillum abundance (A) or sensillum density (B) and are bolded along the *y*‐axis.

**FIGURE 8:**
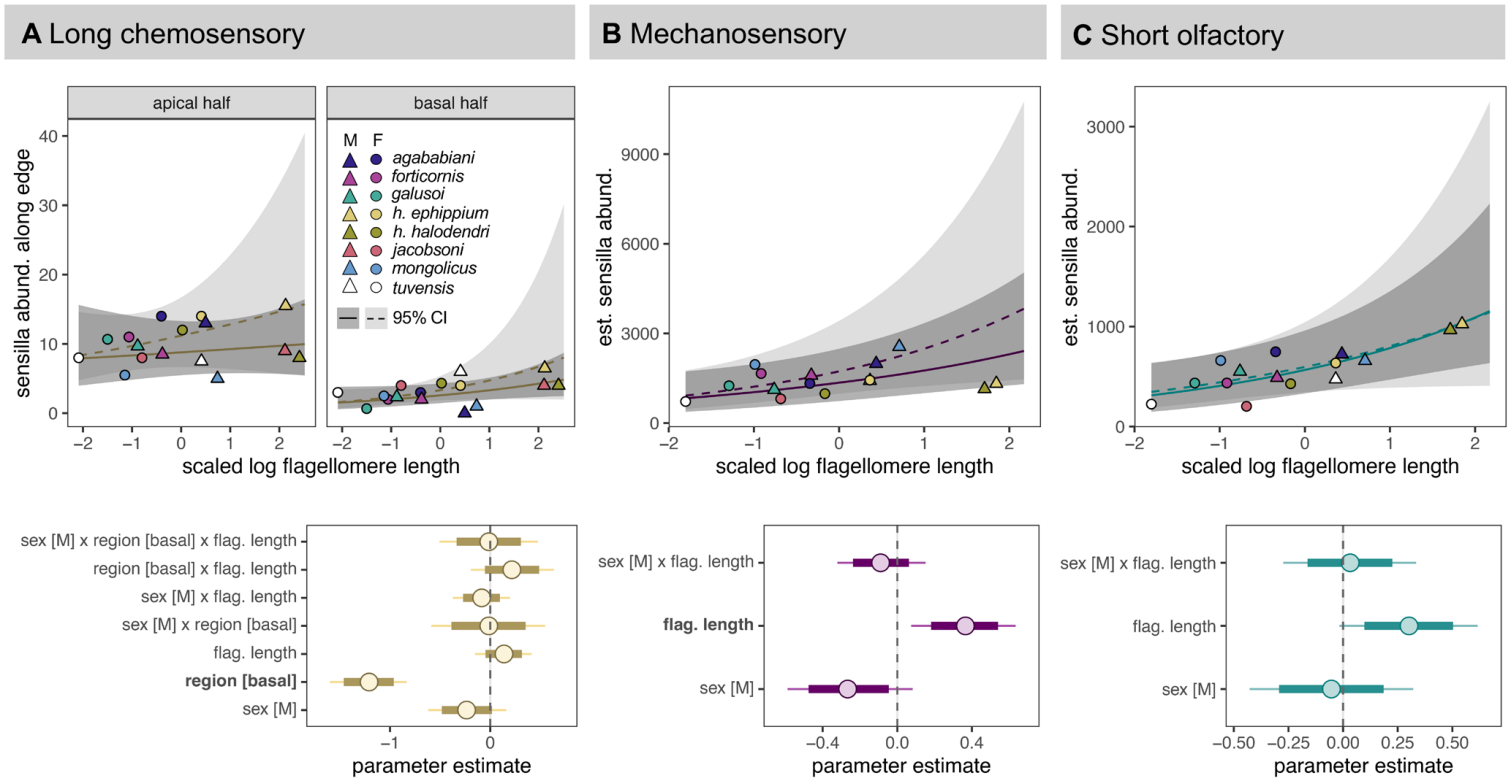
Abundances of sensillum subtypes on the tip antenna segment (f9). Top row shows estimated relationships between sensillum abundance along the edge of the segment (A) or across the surface of the segment (B, C). Ribbons indicate 95% credibility intervals. Dark gray ribbons, solid lines, and triangles are males; light gray ribbons, dashed lines, and circles are females. Point colors indicate taxon. Bottom row shows parameter estimates for each model, with means indicated by circles, 80% credibility intervals indicated by thick lines, and 95% credibility intervals indicated by thin lines. Parameters with 95% credibility intervals that do not overlap 0 affect sensillum abundance and are bolded along the *y*‐axis.

Across species and sexes, sensilla were more abundant and denser distally (i.e., on segments f5 and f9) than proximally (segment f1) (Figure 7). Likewise, long chemosensory hairs were clustered near the tip of f9, with higher counts in the apical half of that segment than the basal half (Figure 8A). Relationships between sensillum abundances and flagellomere length were generally weakly positive (Figures 7A and 8), although flagellomere length did predict total sensillum abundance (Figure 7A) and the abundance of mechanosensory hairs (Figure 8B). However, total sensillum density declined with flagellomere length (Figure 7B), and flagellomere length did not predict the abundance of short olfactory hairs (Figure 8C) or long chemosensory hairs (Figure 8A). In most models, sex or interactions involving sex also failed to predict sensillum abundance. Exceptions include our models of total sensillum abundance and sensillum density, in which males with long flagellomeres were estimated to have lower sensillum abundances (Figure 7A) and lower sensillum densities (Figure 7B) than females with long flagellomeres. In all models, differences among species were attributed more to phylogenetic effects than nonphylogenetic effects, as reflected in the greater effect size of our phylogenetic random intercept than our taxon random intercept (Table S3). However, both effect size estimates had large and consistently overlapping 95% credibility intervals within models, likely owing to substantial variation in sensillum abundance within species. The nested random effect of individual within taxon tended to be weaker than each of the other two random effects.

## 4 Discussion

Biologists have long admired longhorned beetles for their astounding morphological diversity (Pascoe 1864–1869; Linsley 1961; Berry 2008), yet we know surprisingly little about the function and evolution of their most conspicuous trait: their exceptionally elongate antennae. Here, we help bridge this gap by analyzing gross‐ and fine‐scale antenna morphology in *Anoplistes*, a genus with remarkable variability in antenna length and antenna length sexual dimorphism. Looking across species and sexes, we quantified variation in antenna form and tested whether antenna segment length predicted the distribution or abundance of sensory receptors (sensilla) on the antenna surface. Our results can be distilled to three key findings. First, sensilla are clustered near the tips of the antennae, and the abundance of chemosensory hairs is not predicted by antenna length. Second, the most apical antenna segment, f9, is often unusually long relative to other distal segments in *Anoplistes*, especially in males. Taken together, these two results are consistent with the use of elongated male antennae in close‐range mate recognition (the antennal reach hypothesis), as suggested by prior behavioral work on other cerambycids (Hanks et al. 1996a, 1996b). Finally, and somewhat unexpectedly, we found that there is more than one way of producing extremely elongate male antennae in *Anoplistes*: by disproportionately elongating more distal segments, or by roughly equally scaling up the sizes of all antenna segments. Below, we interpret these results in ecological and developmental contexts and suggest future directions for the study of exaggerated antennae.

### 4.1 Insights Into Antenna Function From Fine‐Scale Antenna Morphology

Broadly, our results are more consistent with the antennal reach hypothesis than the olfactory sensitivity hypothesis. Sensillum abundances did not show a consistent pattern of strong positive scaling with antenna length, and although we did find an effect of segment length on total sensillum abundance, this pattern appears to have been driven by mechanosensory sensilla as opposed to chemosensory sensilla. Therefore, elongation of antennae does not increase chemosensory sensillum number as expected by the olfactory sensitivity hypothesis. Instead, sensilla were clustered near the antenna tip, as expected from the antennal reach hypothesis. This interpretation aligns with behavioral studies of other cerambycid taxa—namely, work by Hanks et al. (1996a, 1996b) on a cerambycid species with strong sexual dimorphism in antenna length. In that species, males actively search host logs for mates by walking with their antennae outstretched, only attempting to mate after antennal contact with a female's body (Hanks et al. 1996a). Larger males with longer antennae tend to have higher reproductive success, as they find females faster, providing a potential mechanism for selection to favor greater antennal reach (Hanks et al. 1996b). A similar relationship between antenna length and reproductive success may exist in other cerambycid taxa, as detection of female cuticular hydrocarbons (non‐volatile contact pheromones on the body surface) by males is a necessary component of mate recognition in several cerambycid species (Ginzel et al. 2010). Our study provides an additional morphological line of evidence supporting the importance of contact‐based cues in Cerambycidae. Notably, the high distal abundance of sensilla we see in each of our study species resembles the pattern found in another cerambycid, *Xylotrechus quadripes* Chevrolat, 1863 (Yang et al. 2021), and other (non‐cerambycid) insect taxa that rely heavily on contact chemosensation. Specifically, in diverse species that use their antennae to detect contact pheromones or food (e.g., cockroaches—Norris and Chu 1974; bees—Scheiner et al. 2005; ants—Nakanishi et al. 2009), sensilla are also clustered near the antenna tip. Conversely, species that do not use their antennae for contact chemoreception tend to show different sensillum distributions (Roh et al. 2016; Jayaweera and Barry 2017; Dürr et al. 2022), although there are notable exceptions (e.g., butterflies with olfactory sensilla clustered on the distal club; Donley et al. 2022).

While our work is consistent with Hanks' (Hanks et al. 1996a) hypothesis that elongate cerambycid antennae enable rapid close‐range mate recognition, species of *Anoplistes* also have abundant putative wall and terminal pore hairs, which detect long‐range volatiles (Liu et al. 2012; our study). Thus, detection of host plant chemicals or volatile pheromones may be important to the biology of *Anoplistes*, even if selection on these functions did not favor antenna elaboration in this group. Also worth noting is that our study cannot exclude some other possible functions of exaggerated antennae in *Anoplistes*. Namely, males may use their antennae to chemically recognize and hit rival males during mate‐guarding, as indicated by studies of some cerambycids with strong antenna length dimorphism (Hughes 1981; Hanks et al. 1996a). If *Anoplistes* species also use their antennae in this way, then sensilla may be distally clustered to enable tactile or chemical assessment of opponents, as in ants (Gill et al. 2013).

### 4.2 Evolution of Antenna Form

Within our sample of *Anoplistes*, we found considerable variation in the length and sexual dimorphism of the antennae. There is some evidence that closely related species have similar relative antenna lengths and levels of sexual dimorphism, as lambda was greater than 1 for each trait. However, estimates of phylogenetic signal may be unreliable due to our small taxon sample size (Kamilar and Cooper 2013).

In addition to total antenna lengths, we measured the lengths of individual flagellomeres, revealing two biologically interesting patterns. First, f9—the ninth flagellomere and tip segment—is longer than f8 in males and females of all our focal taxa. The magnitude of this difference varies markedly among sexes and species—more so than for any other pair of adjacent segments. The largest differences occur in males with the longest antennae—specifically, *A. halodendri halodendri*, *A. halodendri ephippium*, and *A. jacobsoni* . A possible explanation for the extreme elongation of f9 in some species is that selection on antennal reach disproportionately targets that segment. In principle, the elongation of any flagellomere should increase antennal reach and be favored by selection. However, it is plausible that the antenna tip is especially important, as it is often the first part of the antenna to contact a novel substrate or potential mate in species with long, flexible contact antennae (Dürr et al. 2022). Lengthening this segment may increase the potential contact area for mechanosensory sensilla spread across its surface, providing an advantage to males searching for mates at close range.

Another factor that may have shaped the unusual morphology of f9 is its unique pattern of development, relative to other flagellomeres. In taxa with nine flagellomeres like *Anoplistes*, all flagellomeres are believed to develop from a single flagellum precursor that repeatedly subdivides (A. Minelli 2004, 2017; Angelini et al. 2009). The first division of this segment forms two segments: f9 and the precursor to all other flagellomeres. This pattern of development suggests that the evolution of f9 may be at least partially decoupled from that of the other flagellomeres, each of which is formed through a larger series of subdivisions. Thus, it may be possible for selection to act with some independence on the last antenna segment, allowing it to exaggerate to extreme proportions.

By comparing male and female flagellomere lengths, we found a second interesting pattern: there are two groups of strongly sexually dimorphic species that express their antenna length dimorphism in different ways. In one pair of non‐sister species (*A. tuvensis* and *A. mongolicus* ), male antennae are scaled‐up versions of female antennae, as in weakly or moderately dimorphic species like *A. galusoi*, *A. forticornis* , or *A. mongolicus* . In the other group (a single clade), males invest disproportionately in more distal segments—particularly f9. The presence of these two patterns in a clade of closely related species suggests that changes in antenna segment allometry can evolve over reasonably short evolutionary timescales. Intriguingly, the alternative pathways to sexual dimorphism we identified align, tentatively, with a potentially relevant ecological difference between taxa: species with scaled‐up male antennae generally mate on smaller host plants (Karpiński, pers. obs.) than do species that show extreme elongation of terminal antenna segments in males (i.e., *A. halodendri ephippium*, *A. halodendri halodendri*, and *A. jacobsoni* ) (Figure 2, Figure S1). In the context of the antennal reach hypothesis, the nature of this difference makes sense. If males rely on their antenna tips to find females at close range, then we might expect stronger selection for antenna tip elongation in males that search for mates across larger areas. We might also expect strong selection on mate‐finding traits (e.g., male antennae) in species with scramble competition mating systems, as members of the more abundant sex must compete for early access to the other sex (Thornhill and Alcock 1983). Although the mating systems of *Anoplistes* species have not yet been described, field observations by one of this study's authors suggest that (1) *A. halodendri* shows male‐biased sex ratios on host plants, particularly near the end of the mating season, and (2) *A. jacobsoni* males actively search their host plants for stationary females (Karpiński, pers. obs.). Both observations are consistent with a pattern of scramble competition in species with elongated, sexually dimorphic antennae. However, additional data are needed to understand whether (and how) the mate‐finding behaviors of these two species differ from *Anoplistes* taxa with shorter, and less sexually dimorphic, antennae.

### 4.3 Phylogeny of *Anoplistes*

To understand the morphology and function of elongated cerambycid antennae, we used a comparative approach involving phylogenetic analyses of new and previously published character data for *Anoplistes*. We also sampled three taxa that were absent from published trees: *A. tuvensis*, *A. agababiani*, and *A. mongolicus* . Below, we consider the phylogenetic impacts of these choices by comparing our summary trees to previous *Anoplistes* phylogenies that were based on different combinations of taxa and characters. Guided by these comparisons, we also discuss how variation in tree topology may impact our understanding of antennal evolution. Finally, we use well‐supported phylogenetic relationships from the literature to justify our taxonomic choices—specifically, our treatment of *A. h. halodendri* and *A. h. ephippium* as independent lineages.

One key difference between our COI‐based consensus trees and previous analyses based on two or three molecular markers (including COI and at least one nuclear marker) is that those other studies have tended to recover *A. halodendri* as sister to *A. galusoi* and/or *A. forticornis* , instead of *A. jacobsoni* (Karpiński et al. 2021, 2023). We did not see such a topology in any of our 100 randomly sampled phylogenetic trees, potentially indicating some discordance between nuclear markers and COI within *Anoplistes*. If *A. halodendri* and *A. jacobsoni* are not sisters, antenna length and antenna sexual dimorphism may be less phylogenetically conserved than our analyses suggest.

Although our summary trees show some topological differences from previous multi‐locus phylogenies, they closely resemble the tree topology recovered by a recent Bayesian (MrBayes), COI‐only study of *Anoplistes* (Karpiński et al. 2024). That tree was based on a larger sample of *Anoplistes* species and specimens than previous work (i.e., Karpiński et al. 2021, 2023), and it showed the same species and subspecies‐level relationships as the mcc tree from our BEAST analysis of COI data, although some nodes were less strongly supported in our tree. Unlike our BEAST tree, though, the Karpiński et al. (2024) phylogeny included multiple specimens per species, as well as several specimens of one subspecies: *A. h. halodendri*. In fact, the COI sequence dataset used in Karpiński et al. (2024) is nearly identical to that used in our MrBayes total evidence analyses, with two important differences: Karpiński et al. (2024) did not include any samples of *A. tuvensis*, but it did include one additional *A. h. halodendri* specimen from a previously unsampled, mountainous portion of that subspecies' range. Another unique feature of our MrBayes analysis is that it was based on both morphological data and COI sequences—a necessity, given that our main goal was to identify the probable phylogenetic placement of two species lacking COI data ( *A. mongolicus* and *A. agababiani*).

Aside from being broadly consistent with the topology of our species‐level summary trees, the results of Karpiński et al. (2024) support our statistical treatment of *A. h. ephippium* and *A. h. halodendri* as distinct lineages. In that study, there was strong support for the reciprocal monophyly of the two subspecies: *A. h. halodendri* was recovered as monophyletic with 100% posterior probability, and the clade of *A. h. halodendri* specimens was sister to the single specimen of *A. h. ephippium* that was sampled. This difference with our results might be due to the inclusion of the “mountain” *A. h. halodendri* specimen—and its recovery at the base of the *A. h. halodendri* clade—by Karpiński et al. (2024). Additionally, that study found weaker COI divergence within *A. h. halodendri* than between *A. h. halodendri* and *A. h. ephippium* (typically < 1% within *A. h. halodendri* vs. ~1.4%–2.4% between subspecies), further supporting the subspecies' distinctness.

Outside Karpiński et al. (2024), there are two other lines of evidence suggesting that *A. h. halodendri* and *A. h. ephippium* are diverging. First, they are geographically isolated, separated by an ~500 km region of Central Kazakhstan that lacks suitable host plants for either subspecies (*Caragana*). This should limit the potential for gene flow among subspecies and (plausibly) enable allopatric divergence. Second, the two subspecies differ in male genitalic characters and body shape, with *A. h. ephippium* appearing elongated relative to *A. h. halodendri*. So stable and apparent are these differences that *A. h. halodendri* and *A. h. ephippium* were originally (in 1817) described as different species and treated as such for more than 150 years (Cherepanov 1990). Thus, available geographic, morphological, and phylogenetic data support the independence of *A. h. halodendri* and *A. h. ephippium* and justify our separate analysis of those two taxa.

## 5 Conclusions

Our work contributes to understanding the function and evolution of a diverse trait in Cerambycidae: their elongate, dimorphic antennae. Collectively, our gross‐ and fine‐scale morphological data from *Anoplistes* reject the olfactory sensitivity hypothesis. Instead, our results are more consistent with the antennal reach hypothesis, which states that elongate, sexually dimorphic antennae are adaptive for close‐range mechano‐ and chemosensation. Although we focused on representatives of a single cerambycid genus, our conclusions may apply to other cerambycids with filiform antennae. In addition to tests of that prediction, we encourage future work on antenna segmentation patterns across a broader sampling of Cerambycidae. Our analyses of these patterns in *Anoplistes* hinted at interesting potential roles for ecology (specifically, the type of host plant used as a mating substrate) and development in the evolution of antenna form. With more species, we could directly test how these factors influence rates and modes of antenna shape evolution, deepening our understanding of an important and diverse functional trait.

## Author Contributions

**Rowan L. K. French:** conceptualization (lead), data curation (lead), formal analysis (lead), funding acquisition (equal), investigation (equal), methodology (equal), project administration (lead), software (lead), validation (lead), visualization (lead), writing – original draft (lead), writing – review and editing (lead). **Magdalena Kowalewska Groszkowska:** investigation (equal), methodology (equal), resources (equal), visualization (supporting), writing – review and editing (supporting). **Locke Rowe:** conceptualization (supporting), funding acquisition (equal), supervision (equal), writing – review and editing (supporting). **D. Luke Mahler:** conceptualization (supporting), funding acquisition (equal), supervision (equal), writing – review and editing (supporting). **Lech Karpiński:** conceptualization (supporting), funding acquisition (equal), investigation (equal), methodology (equal), project administration (supporting), resources (equal), visualization (supporting), writing – review and editing (supporting).

## Conflicts of Interest

The authors declare no conflicts of interest.

## Supporting information


Data S1

## Data Availability

The data, R code, and phylogenetic scripts required to reproduce our analyses are available on Zenodo. https://doi.org/10.5281/zenodo.14642354 (also available at this link: https://zenodo.org/records/14642354).
