# Supplementary material for: Why the Long “Horns”? Fine‐Scale Morphology Suggests Tactile Demands Contributed to the Exaggeration of Male Longhorned Beetle Antennae (Coleoptera: Cerambycidae)
Source: Ecol Evol. 2025 May 8;15(5):e71380. doi: 10.1002/ece3.71380 (PMC12061553; doi:10.1002/ece3.71380)
Supplement: Supplementary file 1 — Data S1 [file ECE3-15-e71380-s001.pdf]

SUPPORTING INFORMATION

Why the long "horns"? Fine-scale morphology suggests tactile demands contributed to the exaggeration of male longhorned beetle antennae (Coleoptera: Cerambycidae)

Supplementary methods

Specimen and habitat photography

Photographs of the habitus were taken with a Canon EOS 50D digital camera equipped with a Canon 100 mm f/2.8 USM Macro lens and a Canon MP-E 65 mm f/2.8 1–5×lens. The images produced were stacked, aligned and combined using Helicon Focus (www.heliconsoft.com) and Zerene Stacker (www.zerenesystems.com) software. Some images were taken and automatically stacked and combined using a Keyence VHX-7000 digital microscope at MIZ. Photographs of the cerambycids in situ, their host plants and habitats were taken with a Canon EOS 600D and a Nikon Coolpix AW110 cameras. All plates were prepared using GIMP v. 2.10.10 (www.gimp.org).

Table S1. Morphological characters used in MrBayes phylogenetic analysis

Trait	Category	Description	States
1	Body size	Average body size	(0) small; (1) medium; (2) large; (3) very large
2	Body shape	Overall body thickness	(0) slim; (1) moderate; (2) corpulent
3	Pronotum	Pronotal punctation	(0) wide and distinct, regular, honeycomb-like; (1) fine and sparse, rather regular, with clear space between punctures; (2) fine and dense, rather regular, without clear space between punctures; (3) irregular and shallow; (4) fine and dense, irregular, with irregular space between punctures
4	Pronotum	Pronotal lateral tubercles	Pronotal lateral tubercles: (0) rather indistinct – pronotum almost rounded; (1) slight; (2) pronounced
5	Elytra	Ratio of elytral length to width in males	(0) 1.9–2.15; (1) 2.2–2.45; (2) 2.5–2.65; (3) > 2.7
6	Underside	Prosternal process	(0) cross-shaped; (1) clearly bifurcated at the end; (2) short and broadly ended; (3) lobate; (4) long and tongue-shaped
7	Tarsi	Ratio of metatarsomere 1 to metatarsomere 2 and 3 combined in males	(0) 1–1.1; (1) 1.11–1.2; (2) 1.3–1.4; (3) >1.4
8	Tarsi	Ratio of length to width (at widest point) of metatarsomere 2	(0) 0.95–1.05; (1) 1.45–1.55; (2) 1.65–1.75; (3) >1.95
9	Male genitalia	Overall shape of lateral lobes of tegmen	(0) short, very robust, parallel sided; (1) relatively long, robust, tapering at ends; (2) rather short and robust, with parallel tips but strongly tapering overall; (3) relatively short and thin, rather parallel sided; (4) long, thin, parallel sided
10	Male genitalia	Lateral lobes apex shape	(0) wide and blunt; (1) broadly rounded; (2) bevelled on outer edge, rounded inwards; (3) rounded outwards, with acute angles inwards
11	Male genitalia	Lateral lobes apex hairs	(0) few, sparse and short, recumbent and curly; (1) few, sparse and moderately long, protruding and straight; (2) relatively abundant and dense, short, protruding and straight; (3) few, sparse and long, protruding and slightly curly
12	Male genitalia	Indentation between lateral lobes of tegmen	(0) to about 1/3 of their length; (1) to about 1/2 of their length; (2) to about 2/3 of their length
13	Pronotum	Pronotal lateral spines	(0) absent; (1) present

Table S2. Morphological character matrix used in MrBayes total evidence phylogenetic analysis

Trait	*A. agababiani*	*A. forticornis*	*A. galusoi*	*A. mongolicus*	*A. halodendri ephippium*	*A. halodendri halodendri*	*A. jacobsoni*	*A. tuvensis*	*Purpuricenus kaehleri*
1	1	1	1	2	1	1	1	0	3
2	1	2	1	2	0	1	0	0	2
3	1	2	1	3	0	0	0	1	4
4	1	1	0	0	1	1	1	2	2
5	1	1	2	1	3	3	3	2	0
6	1	4	4	0	2	2	3	2	–
7	1	2	3	0	1	1	1	1	0
8	1	0	0	1	2	2	3	1	0
9	0	3	3	1	4	2	2	3	4
10	0	1	1	2	1	3	3	1	3
11	0	2	2	0	1	1	1	3	1
12	0	2	2	2	2	0	0	1	1
13	0	0	0	0	0	0	0	0	1

Table S3. Model results from brms. Results are based on posterior distributions generated by pooling across 100 iterations involving different phylogenetic trees. Effect types labeled "Main" are population parameters (slopes and intercepts); "Rand" indicates random intercept effects; "Shape" indicates parameters related to the shape of the response variable distribution. l-95% CI and u-95% CI are the lower and upper bounds of the 95% credibility interval, respectively. ESS is effective sample size.

Model	Effect	Estimate	Est. error	l-95% CI	u-95% CI	Rhat	Bulk ESS	Tail ESS	Effect type
**Segment area**	Intercept	13.08	0.19	12.71	13.49	1	96698	74962	Main
	sexM	-0.04	0.05	-0.13	0.06	1	990581	1279771	Main
	segment.5	0.06	0.05	-0.03	0.15	1	513872	899203	Main
	segment.9	-0.3	0.05	-0.39	-0.21	1	543053	935056	Main
	log_antennal_seg_length	0.58	0.05	0.48	0.69	1	472704	824192	Main
	sexM:segment.5	-0.13	0.05	-0.23	-0.04	1	825139	1185095	Main
	sexM:segment.9	-0.1	0.06	-0.22	0.01	1	651144	1041390	Main
	sexM:log_antennal_seg_length	-0.04	0.05	-0.15	0.06	1	1010172	1280148	Main
	segment.5:log_antennal_seg_length	-0.13	0.04	-0.2	-0.06	1	1328338	1381522	Main
	segment.9:log_antennal_seg_length	-0.25	0.03	-0.32	-0.18	1	1491801	1443430	Main
	sexM:segment.5:log_antennal_seg_length	0.05	0.05	-0.06	0.14	1	1119788	1311322	Main
	sexM:segment.9:log_antennal_seg_length	0.05	0.05	-0.05	0.15	1	989205	1261655	Main
	phylo	0.27	0.16	0.02	0.65	1.01	38356	141410	Rand
	taxon	0.17	0.13	0.01	0.48	1	91550	948195	Rand
	taxon:individual	0.09	0.02	0.05	0.14	1	342539	612309	Rand
	sigma	0.08	0.01	0.06	0.09	1	556712	866993	Shape
**Total sensillum abundance**	Intercept	5.63	0.25	5.15	6.14	1	160476	102218	Main
	sexM	-0.17	0.12	-0.41	0.08	1	1532285	1471553	Main
	segment.5	1.74	0.13	1.49	1.99	1	1083249	1355432	Main
	segment.9	1.75	0.13	1.49	2.01	1	1143289	1374491	Main
	log_antennal_seg_length	0.26	0.13	0.02	0.51	1	942685	1205818	Main
	sexM:segment.5	-0.07	0.16	-0.39	0.24	1	1274016	1457159	Main
	sexM:segment.9	-0.06	0.18	-0.41	0.29	1	1236616	1447983	Main
	sexM:log_antennal_seg_length	-0.36	0.14	-0.64	-0.08	1	1122385	1405940	Main
	segment.5:log_antennal_seg_length	0.08	0.13	-0.18	0.33	1	1384641	1503496	Main
	segment.9:log_antennal_seg_length	-0.04	0.13	-0.3	0.21	1	1356263	1465459	Main
	sexM:segment.5:log_antennal_seg_length	0.3	0.18	-0.06	0.66	1	1202584	1448060	Main

	sexM:segment.9:log_antennal_seg_length	0.33	0.18	-0.02	0.68	1	1117974	1404595	Main
	phylo	0.32	0.21	0.02	0.83	1	56124	188095	Rand
	taxon	0.22	0.17	0.01	0.62	1	167666	911002	Rand
	taxon:individual	0.11	0.06	0.01	0.24	1	434391	722298	Rand
	shape	12.62	2.31	8.65	17.69	1	1032063	1406907	Shape
**Sensillum density**	Intercept	-7.47	0.11	-7.69	-7.24	1	1102585	558864	Main
	sexM	-0.05	0.11	-0.27	0.17	1	1370671	1503234	Main
	segment.5	1.66	0.1	1.46	1.86	1	1285132	1487943	Main
	segment.9	2.03	0.11	1.82	2.24	1	1292972	1480380	Main
	log_antennal_seg_length	-0.37	0.1	-0.56	-0.17	1	1043841	1351074	Main
	sexM:segment.5	0.02	0.14	-0.25	0.31	1	1193615	1444376	Main
	sexM:segment.9	0.02	0.15	-0.28	0.32	1	1199692	1450463	Main
	sexM:log_antennal_seg_length	-0.3	0.14	-0.57	-0.03	1	972417	1332873	Main
	segment.5:log_antennal_seg_length	0.21	0.11	-0.01	0.44	1	1173112	1430034	Main
	segment.9:log_antennal_seg_length	0.21	0.11	-0.02	0.43	1	1129594	1400184	Main
	sexM:segment.5:log_antennal_seg_length	0.25	0.17	-0.08	0.58	1	1050808	1388074	Main
	sexM:segment.9:log_antennal_seg_length	0.29	0.16	-0.03	0.61	1	996857	1345077	Main
	phylo	0.11	0.09	0	0.33	1	67012	163112	Rand
	taxon	0.08	0.07	0	0.25	1	755981	965225	Rand
	taxon:individual	0.08	0.05	0	0.17	1	517200	739023	Rand
	shape	20.45	4.69	12.91	31.19	1	1314306	1455892	Shape
**Long chemo. abund.**	Intercept	2.4	0.23	1.9	2.82	1	789426	255777	Main
	regionbasalhalf	-1.21	0.2	-1.6	-0.83	1	1556192	1549657	Main
	sexM	-0.23	0.2	-0.62	0.16	1	1457033	1482372	Main
	log_antennal_seg_length	0.13	0.14	-0.15	0.41	1	1109978	1320422	Main
	regionbasalhalf:sexM	-0.02	0.29	-0.59	0.55	1	1917121	1576528	Main
	regionbasalhalf:log_antennal_seg_length	0.22	0.21	-0.19	0.63	1	1379015	1507583	Main
	sexM:log_antennal_seg_length	-0.09	0.14	-0.37	0.2	1	1388217	1452629	Main
	regionbasalhalf:sexM:log_antennal_seg_length	-0.01	0.25	-0.51	0.48	1	1502663	1518350	Main
	phylo	0.23	0.18	0.01	0.68	1	60334	280769	Rand
	taxon	0.16	0.14	0.01	0.5	1	775156	1015919	Rand
	taxon:individual	0.11	0.08	0	0.29	1	844114	964016	Rand
	shape	77.84	69.21	12.48	266.86	1	2710956	1645922	Shape
**Mechano. abund.**	Intercept	7.47	0.31	6.89	8.14	1	837096	160056	Main
	sexM	-0.26	0.17	-0.59	0.08	1	1014216	1161601	Main

	log_antennal_seg_length	0.36	0.14	0.07	0.63	1	935542	1050405	Main
	sexM:log_antennal_seg_length	-0.09	0.12	-0.32	0.15	1	1530887	1339552	Main
	phylo	0.4	0.26	0.03	1.01	1	65877	151760	Rand
	taxon	0.26	0.2	0.01	0.75	1	155809	837184	Rand
	shape	19.63	6.45	9.22	34.23	1	1097010	1197493	Shape
**Short olfactory abund.**	Intercept	6.39	0.28	5.84	6.95	1	914846	384771	Main
	sexM	-0.05	0.19	-0.43	0.32	1	1354666	1430482	Main
	log_antennal_seg_length	0.3	0.16	-0.02	0.62	1	1114862	1260697	Main
	sexM:log_antennal_seg_length	0.03	0.15	-0.27	0.33	1	1450522	1393663	Main
	phylo	0.3	0.24	0.01	0.9	1	130783	465518	Rand
	taxon	0.23	0.19	0.01	0.69	1	571755	855358	Rand
	shape	10.79	3.46	5.27	18.7	1	1206944	1335267	Shape

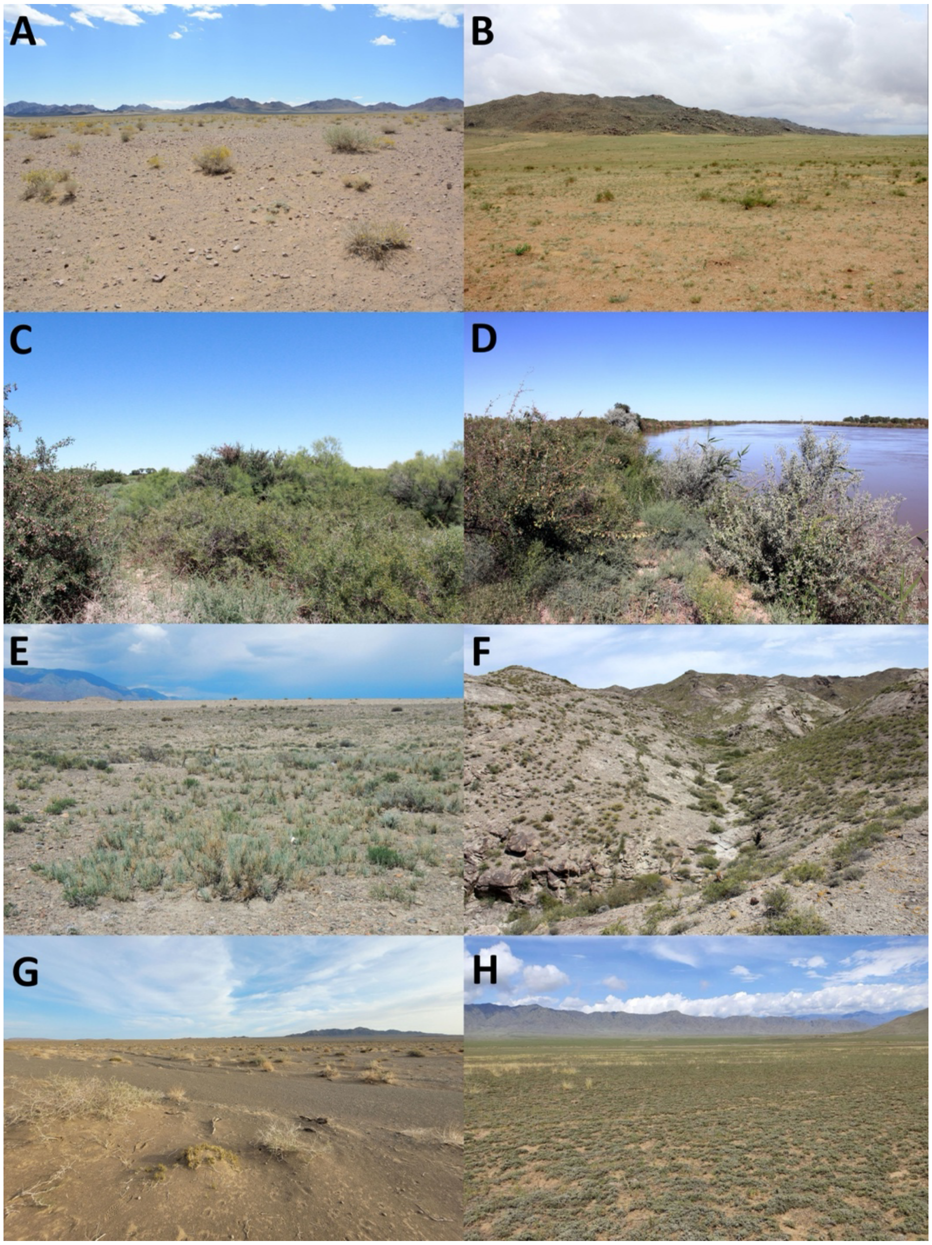

Fig. S1. Habitats of the studied *Anoplistes* taxa. (A, B) *Anoplistes halodendri halodendri* (different ecotypes; semi-desert and steppe habitat with *Caragana* in NW and E Mongolia, respectively); (C, D) *Anoplistes jacobsoni* (tugay habitat with *Halimodendron* in S Kazakhstan); (E) *Anoplistes forticornis* (habitat near a lake shore with *Ephedra* in N Kyrgyzstan); (F) *Anoplistes galusoi* (mountain slopes overgrown with *Ephedra* in SE Kazakhstan; (G) *Anoplistes mongolicus* (semi-desert habitat with *Calligonum* in SW Mongolia); (H) *Anoplistes tuvensis* (*Nanophyton* "desert" habitat in S Russia).

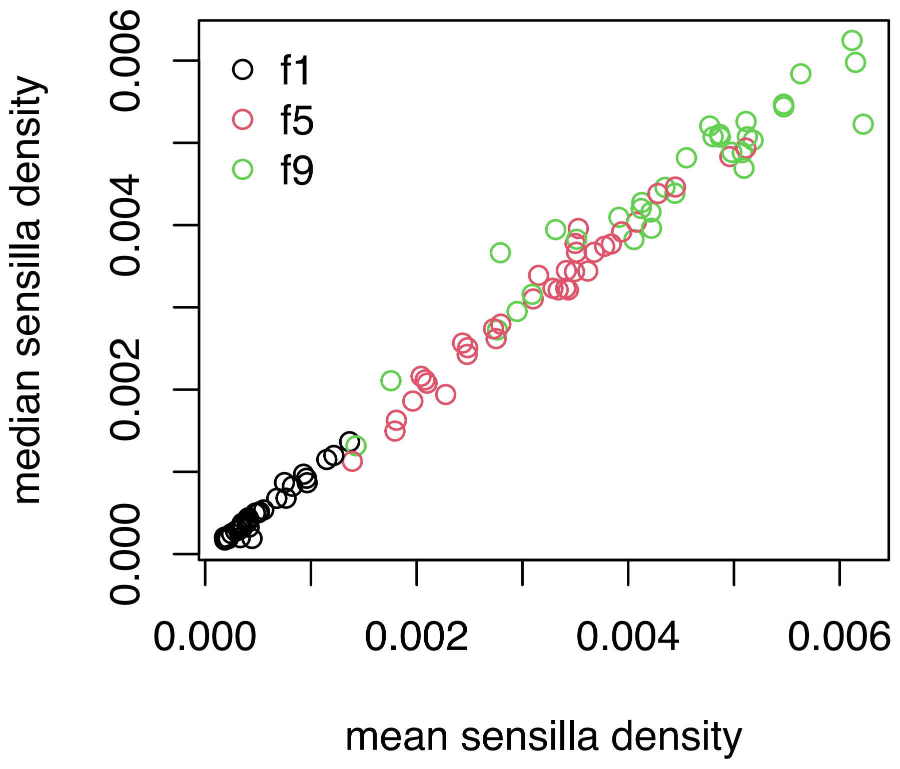

Fig. S2. Correlation between mean and median sensilla densities. Points represent individual-level data for flagellomeres 1 (black), 5 (pink), or 9 (green).

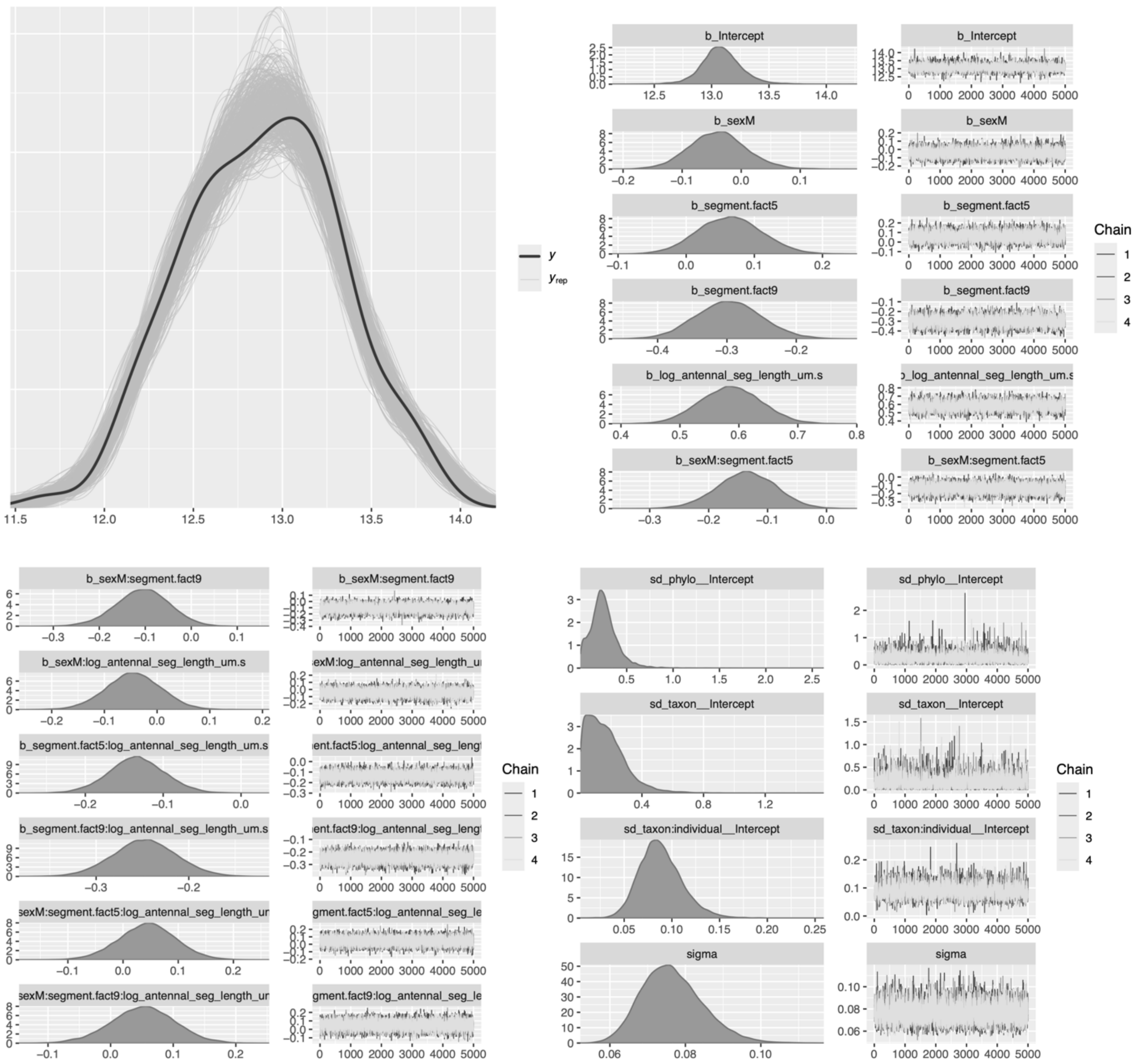

Fig. S3. Segment area model: posterior predictive checks based on 500 random draws from model results that were pooled across 100 iterations (top left) and trace plots for a randomly selected iteration. In the posterior predictive plot, *y* represents our observed response data, while each y_{rep} line represents a replicated response dataset that was simulated under our model. The remaining subfigures show model estimates for each parameter across 5000 post-burnin samples and four chains (left: distribution of model estimates after pooling all four chains and samples; right: fuzzy caterpillar plots showing individual sample estimates, with chains overlaid).

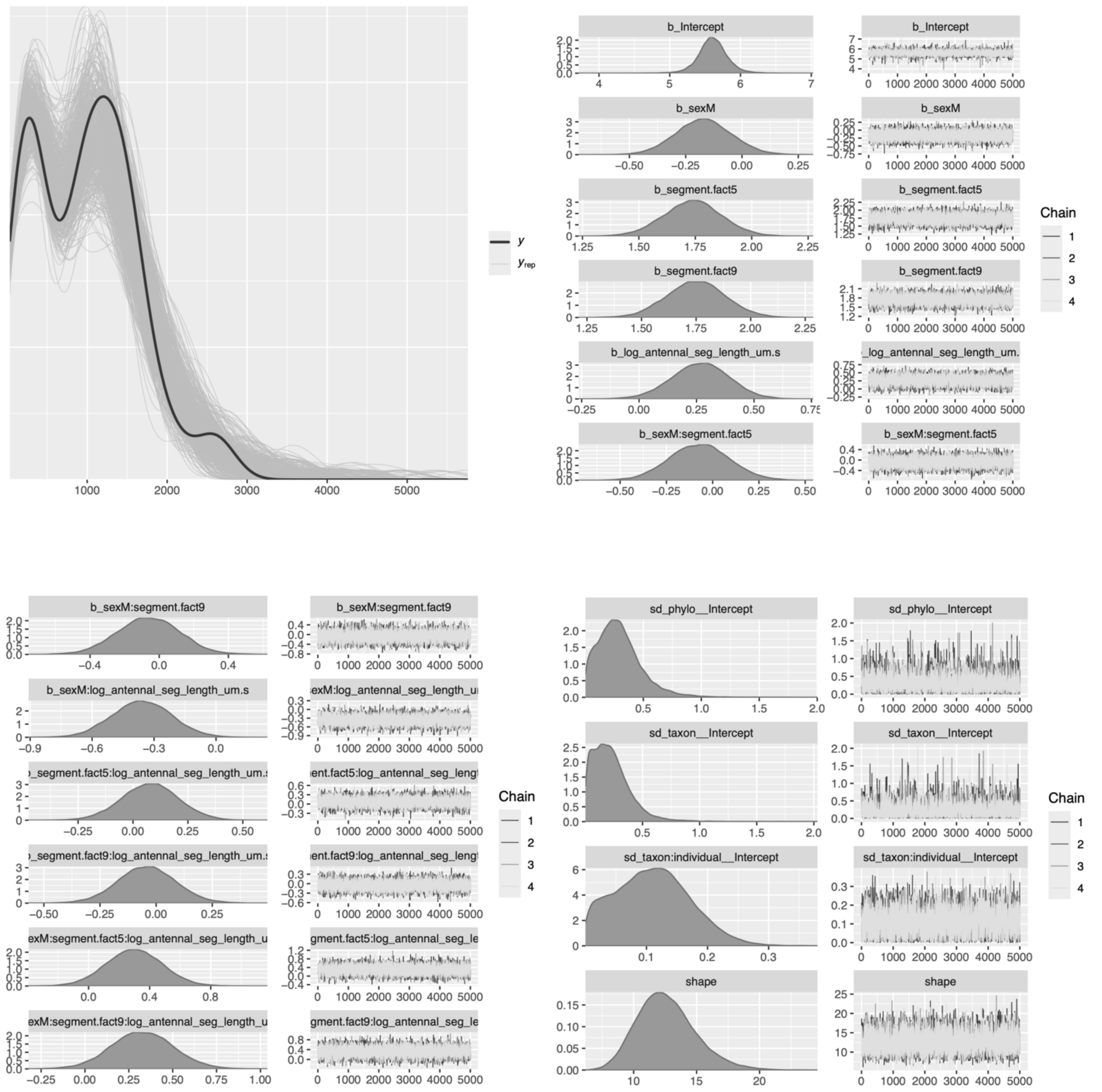

Fig. S4. Total sensilla abundance model: posterior predictive checks based on 500 random draws from model results that were pooled across 100 iterations (top left) and trace plots for a randomly selected iteration. In the posterior predictive plot, *y* represents our observed response data, while each y_{rep} line represents a replicated response dataset that was simulated under our model. The remaining subfigures show model estimates for each parameter across 5000 post-burnin samples and four chains (left: distribution of model estimates after pooling all four chains and samples; right: fuzzy caterpillar plots showing individual sample estimates, with chains overlaid).

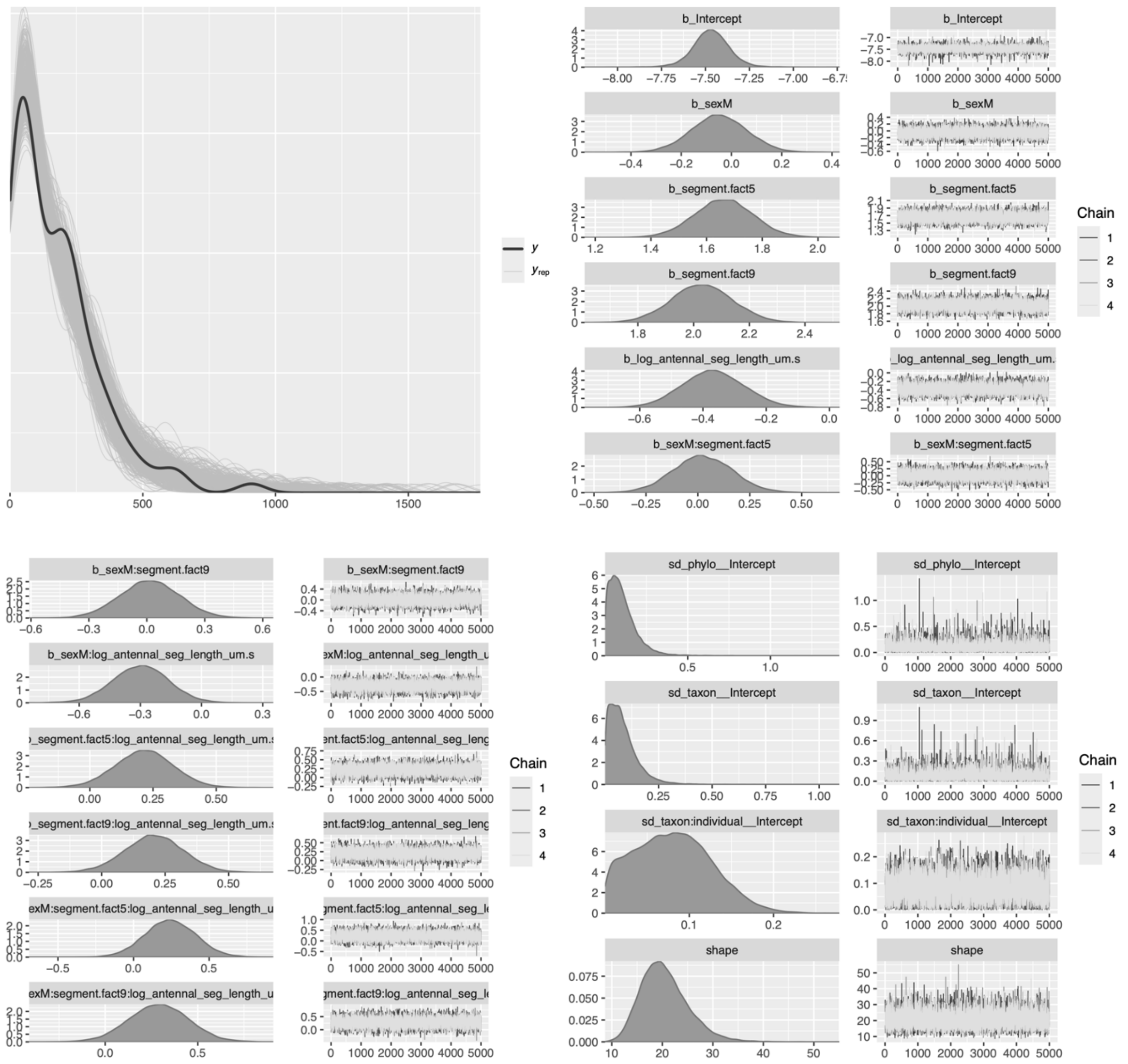

Fig. S5. Total sensilla density model: posterior predictive checks based on 500 random draws from model results that were pooled across 100 iterations (top left) and trace plots for a randomly selected iteration. In the posterior predictive plot, *y* represents our observed response data, while each y_{rep} line represents a replicated response dataset that was simulated under our model. The remaining subfigures show model estimates for each parameter across 5000 post-burnin samples and four chains (left: distribution of model estimates after pooling all four chains and samples; right: fuzzy caterpillar plots showing individual sample estimates, with chains overlaid).

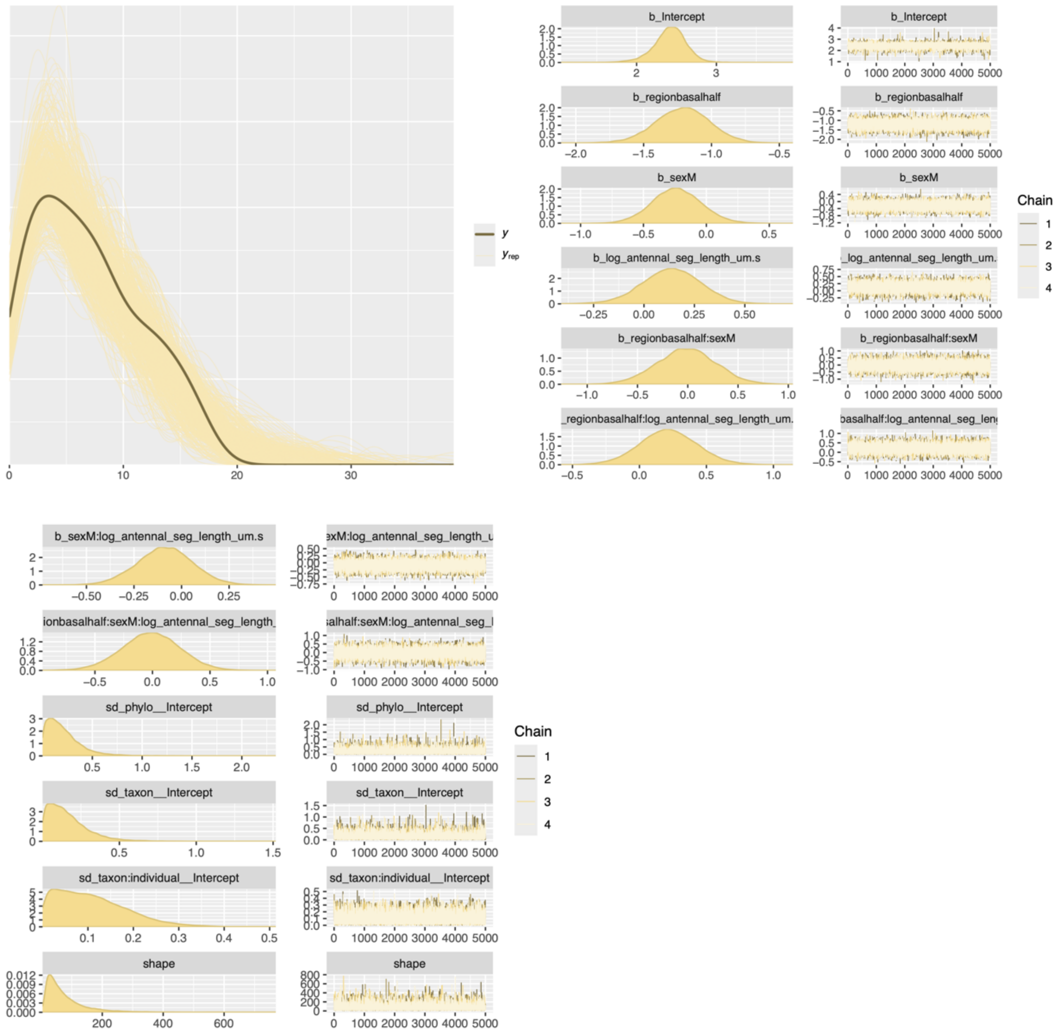

Fig. S6. Long chemosensory hair model: posterior predictive checks based on 500 random draws from model results that were pooled across 100 iterations (top left) and trace plots for a randomly selected iteration. In the posterior predictive plot, *y* represents our observed response data, while each y_{rep} line represents a replicated response dataset that was simulated under our model. The remaining subfigures show model estimates for each parameter across 5000 post-burnin samples and four chains (left: distribution of model estimates after pooling all four chains and samples; right: fuzzy caterpillar plots showing individual sample estimates, with chains overlaid).

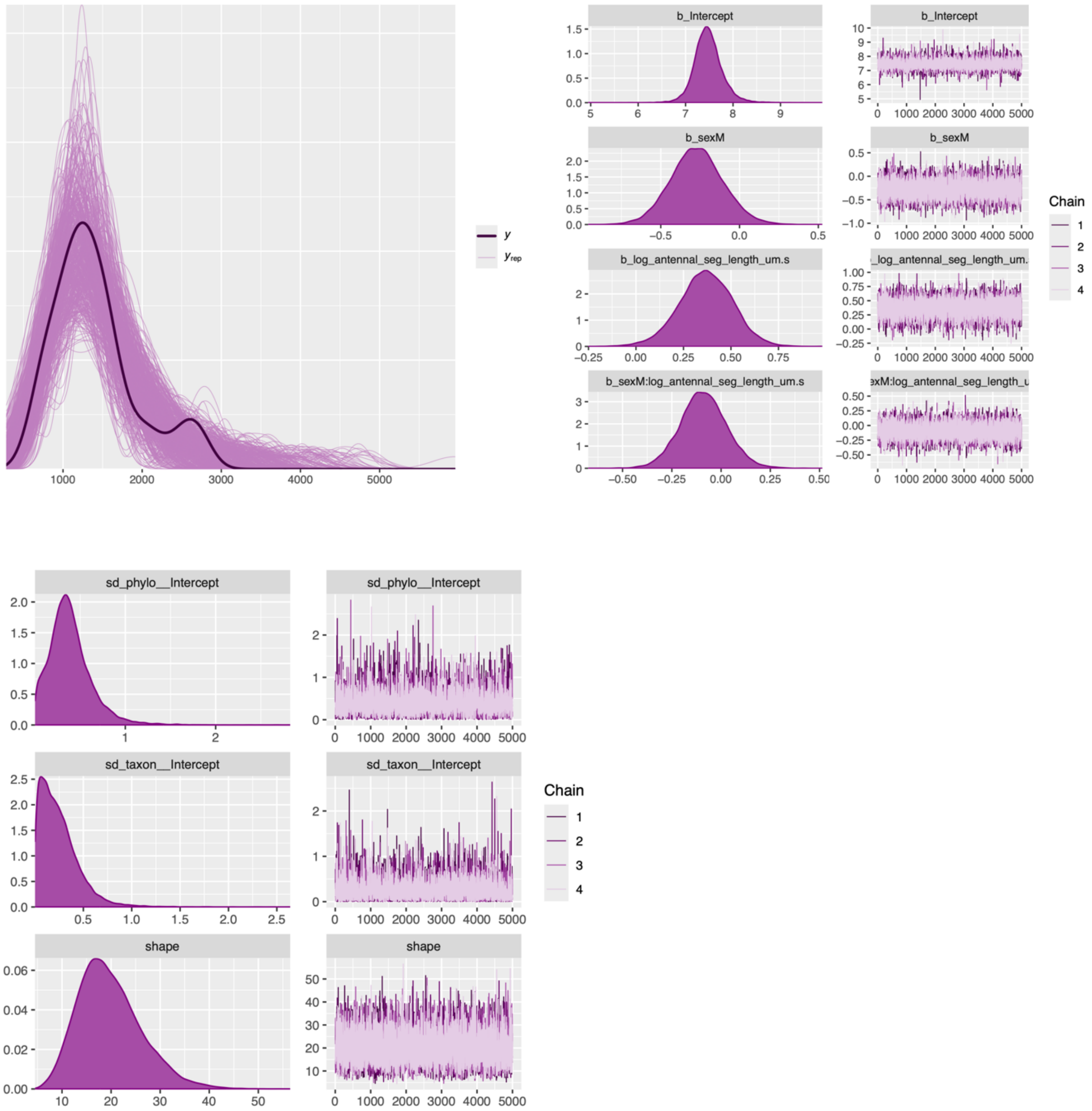

Fig. S7. Mechanosensory hair model: posterior predictive checks based on 500 random draws from model results that were pooled across 100 iterations (top left) and trace plots for a randomly selected iteration. In the posterior predictive plot, *y* represents our observed response data, while each y_{rep} line represents a replicated response dataset that was simulated under our model. The remaining subfigures show model estimates for each parameter across 5000 post-burnin samples and four chains (left: distribution of model estimates after pooling all four chains and samples; right: fuzzy caterpillar plots showing individual sample estimates, with chains overlaid).

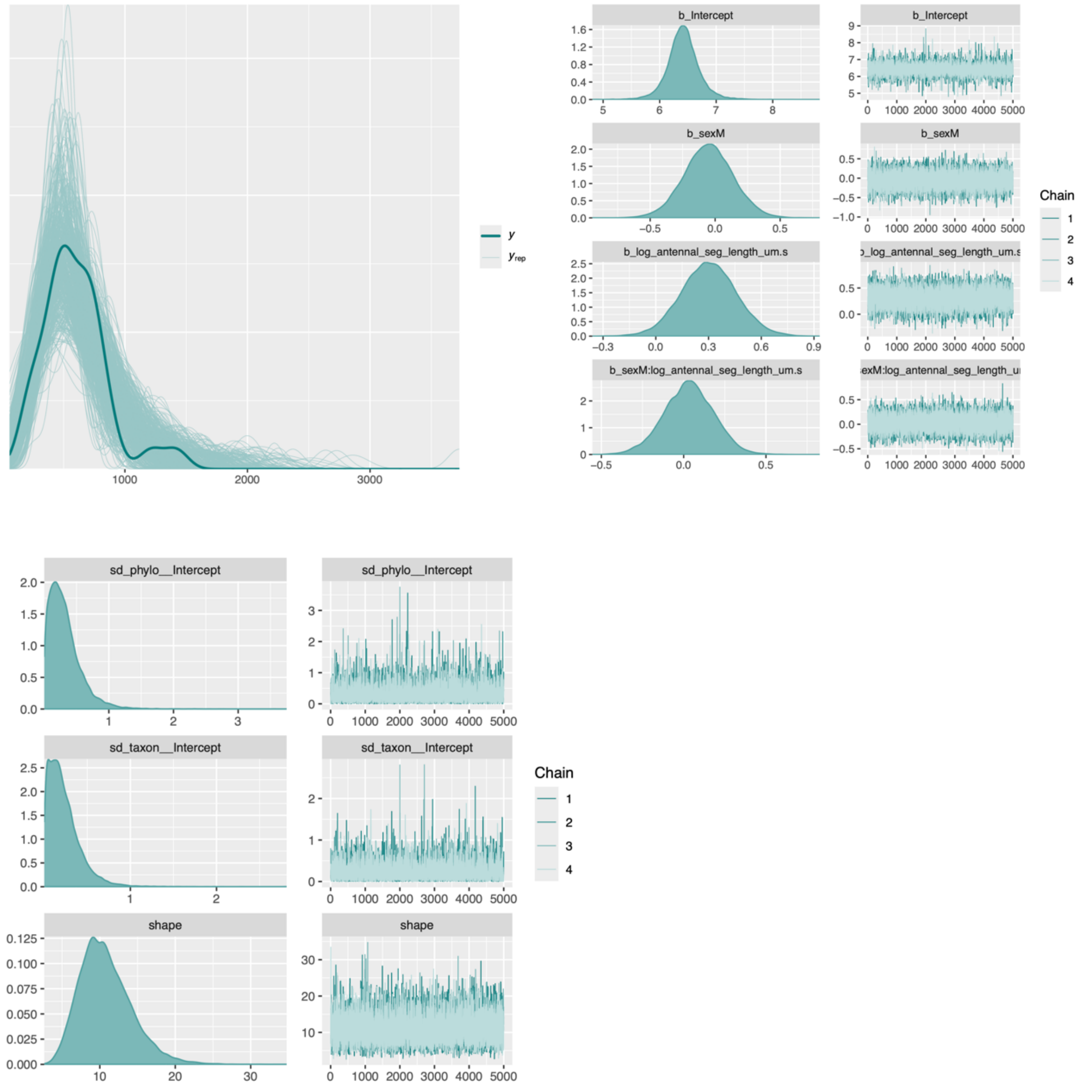

Fig. S8. Short olfactory hair model: posterior predictive checks based on 500 random draws from model results that were pooled across 100 iterations (top left) and trace plots for a randomly selected iteration. In the posterior predictive plot, *y* represents our observed response data, while each y_{rep} line represents a replicated response dataset that was simulated under our model. The remaining subfigures show model estimates for each parameter across 5000 post-burnin samples and four chains (left: distribution of model estimates after pooling all four chains and samples; right: fuzzy caterpillar plots showing individual sample estimates, with chains overlaid).

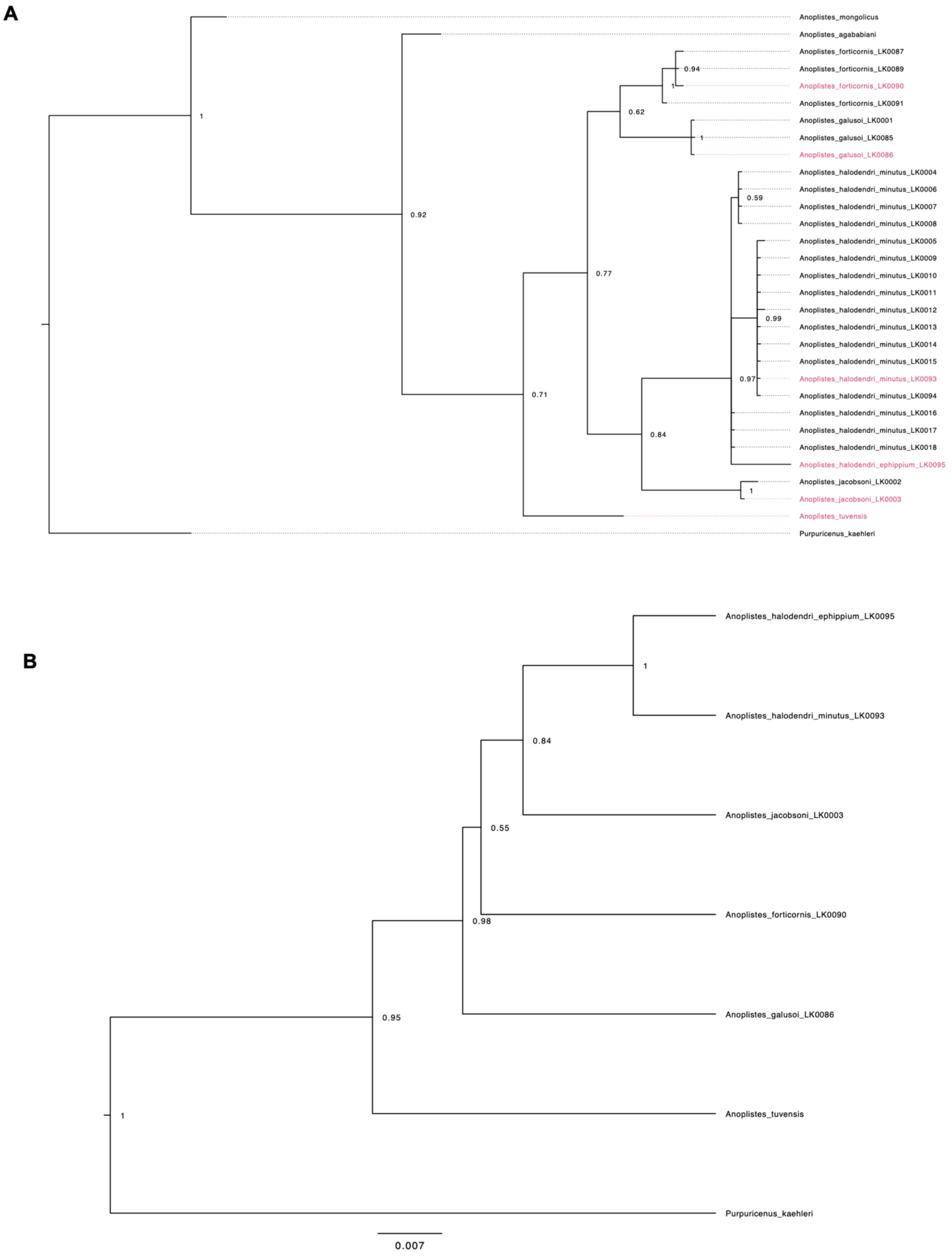
A
Anoplistes_mongolicus
Anoplistes_agababiani
Anoplistes_forticornis_LK0087
Anoplistes_forticornis_LK0089
Anoplistes_forticornis_LK0090
Anoplistes_forticornis_LK0091
Anoplistes_galusoi_LK0001
Anoplistes_galusoi_LK0085
Anoplistes_galusoi_LK0086
Anoplistes_halodendri_minutus_LK0004
Anoplistes_halodendri_minutus_LK0006
Anoplistes_halodendri_minutus_LK0007
Anoplistes_halodendri_minutus_LK0008
Anoplistes_halodendri_minutus_LK0005
Anoplistes_halodendri_minutus_LK0009
Anoplistes_halodendri_minutus_LK0010
Anoplistes_halodendri_minutus_LK0011
Anoplistes_halodendri_minutus_LK0012
Anoplistes_halodendri_minutus_LK0013
Anoplistes_halodendri_minutus_LK0014
Anoplistes_halodendri_minutus_LK0015
Anoplistes_halodendri_minutus_LK0093
Anoplistes_halodendri_minutus_LK0094
Anoplistes_halodendri_minutus_LK0016
Anoplistes_halodendri_minutus_LK0017
Anoplistes_halodendri_minutus_LK0018
Anoplistes_halodendri_ephippium_LK0095
Anoplistes_jacobsoni_LK0002
Anoplistes_jacobsoni_LK0003
Anoplistes_tuvensis
Purpuricenus_kaehleri
1
0.92
0.94
1
0.62
1
0.59
0.77
0.99
0.71
0.97
0.84
1
B
Anoplistes_halodendri_ephippium_LK0095
Anoplistes_halodendri_minutus_LK0093
Anoplistes_jacobsoni_LK0003
Anoplistes_forticornis_LK0090
Anoplistes_galusoi_LK0086
Anoplistes_tuvensis
Purpuricenus_kaehleri
1
0.84
0.55
0.98
0.95
1
0.007

Fig. S9. Bayesian phylogenies of *Anoplistes*. (A) 50% majority-rule consensus tree (total evidence tree) based on analysis of morphological and molecular data in MrBayes. (B) Maximum clade credibility (mcc) tree reconstructed via BEAST analysis of a COI-only alignment. Node labels in both trees are posterior probabilities. Ingroup tips coloured pink in (A) were also used in (B). Vouchers labeled *Anoplistes halodendri minutus* correspond to what we refer to as *A. halodendri halodendri* elsewhere in our manuscript. Our general use of the name *A. halodendri halodendri* reflects new (but not yet published) information about the type material of this taxon. Given this new information, we wished to avoid spreading *A. halodendri minutus* as a potentially erroneous name. Here, we made an exception by presenting original voucher names. We did this for transparency, and to facilitate comparison to prior work. Both plots were created using FigTree.

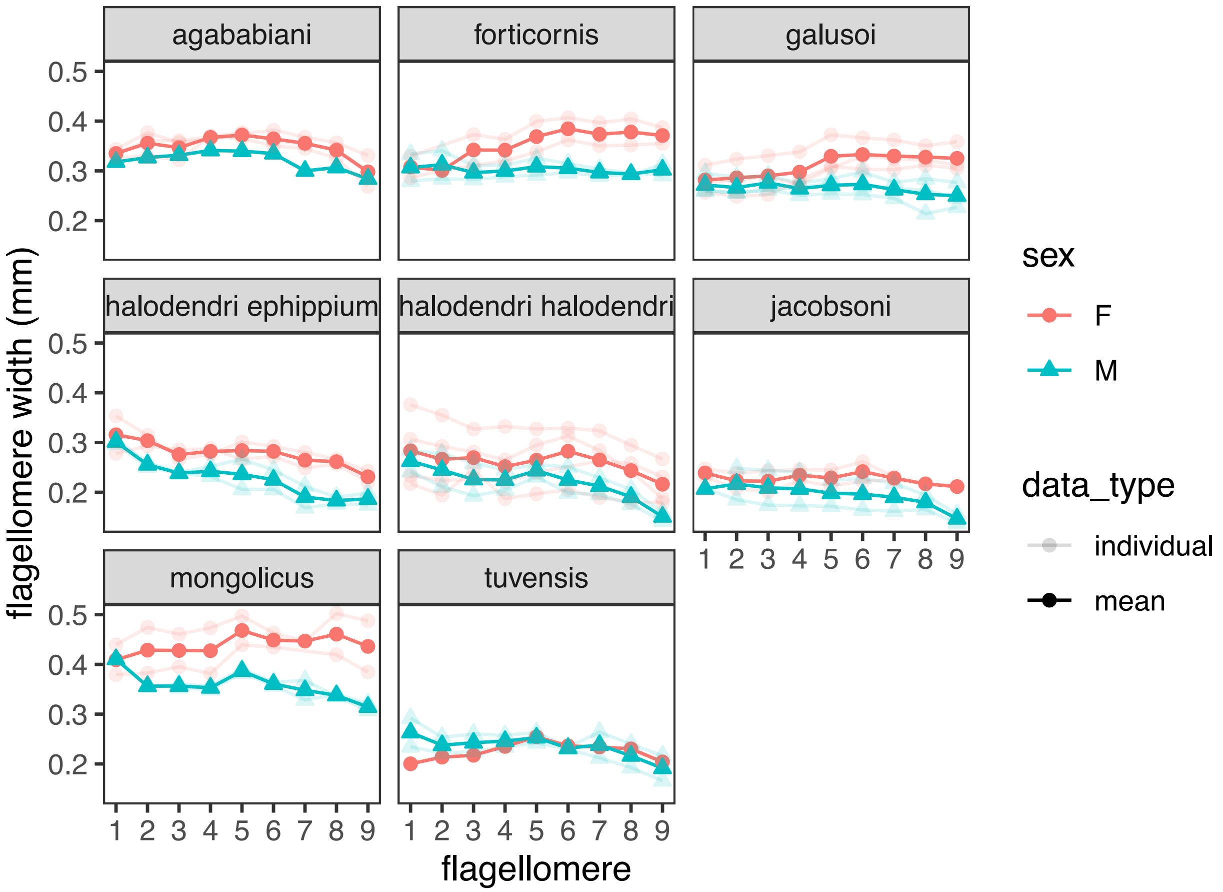

Fig. S10. Flagellomere widths plotted against flagellomere position. flagellomere 1 is the most proximal flagellomere; flagellomere 9 is the antennal tip (most distal flagellomere). Sex and taxon-specific means are plotted (triangles, circles, and lines with no transparency) along with raw, individual-level data (partial transparency).

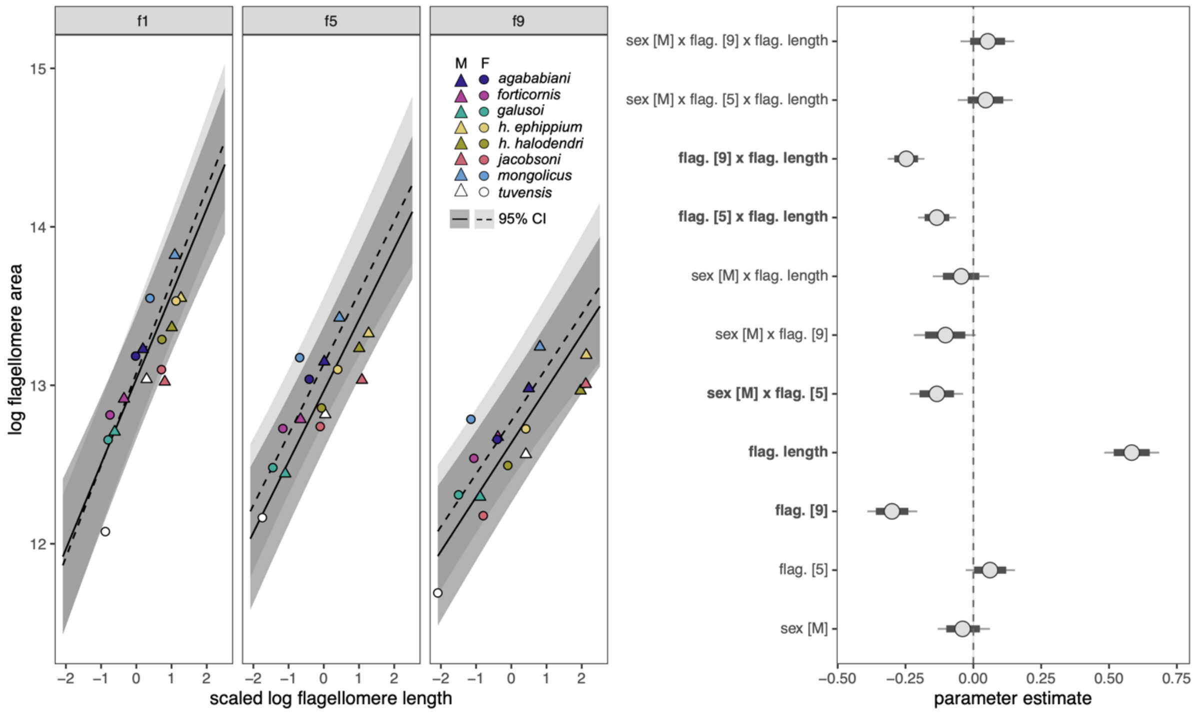

Fig. S11. Fitted brms model of flagellomere area. The left panel is faceted by segment. Dark grey ribbons, triangles, and solid regression lines indicate males; light grey ribbons, circles, and dashed regression lines indicate females. Ribbons are 95% credibility intervals. Points represent means for distinct taxa and sexes and colours represent taxon identity. Right panel shows parameter estimates from the brms model; circles are means, thick lines are 80% credibility intervals, and thin lines are 95% credibility intervals. Parameters with 95% credibility intervals that do not overlap zero are bolded.
